# Nurse Performance Evaluation in Healthcare: A Systematic Review of Measurement Approaches, Determinants, Appraisal Processes, and Outcomes

**DOI:** 10.1155/jonm/9209587

**Published:** 2026-10-12

**Authors:** Ali Rowshanbakht, Masoud Etedali, Ali Valinejadi, Ashraf Salehi, Hamid Momeni, Meghdad Rahati

**Affiliations:** ^1^ Department of Nursing Shahid Sadoughi University of Medical Sciences Yazd Iran ssu.ac.ir; ^2^ Department of Medical-Surgical Nursing Khomein University of Medical Sciences Khomeyn Iran; ^3^ Department of Health Information Management Khomein University of Medical Sciences Khomeyn Iran; ^4^ Department of Healthcare Services Management Khomein University of Medical Sciences Khomeyn Iran

**Keywords:** clinical competence, nurse performance, nursing management, performance appraisal, performance evaluation, systematic review

## Abstract

**Background:**

Nurse performance evaluation encompasses multiple related but distinct activities, including measurement of work performance or professional competence and formal organizational appraisal processes. However, considerable conceptual and methodological heterogeneity exists across the literature, and the relationships among measurement approaches, determinants of measured performance, formal appraisal processes, and appraisal‐related outcomes remain unclear.

**Aim:**

To systematically synthesize primary empirical evidence on nurse performance evaluation in clinical healthcare settings and develop an evidence‐informed conceptual framework integrating measurement approaches, determinants, formal appraisal processes, and appraisal‐related outcomes.

**Methods:**

A systematic search of PubMed, Scopus, Web of Science, CINAHL, and Embase was conducted for studies published between January 2010 and November 2025. Forty primary empirical studies met the eligibility criteria and were included in the qualitative synthesis. Methodological quality was assessed using design‐appropriate critical appraisal approaches, including the Mixed Methods Appraisal Tool (MMAT, 2018) for applicable study designs and appropriate methodological criteria for instrument‐development, Delphi, decision‐analysis, model‐development, and framework‐development studies. Findings were synthesized using the thematic synthesis approach proposed by Thomas and Harden (2008).

**Results:**

Forty primary empirical studies published between 2010 and 2025 were included. The evidence was conceptually heterogeneous and contributed to four overlapping domains: tools and methods for measuring nurse performance or clinically relevant professional competence; individual, professional, organizational, and work‐environment determinants associated with measured performance; formal performance‐appraisal processes and systems; and outcomes examined directly in relation to appraisal processes. Sixteen studies (40.0%) contributed to the tools/measurement domain, 19 (47.5%) examined determinants of measured nurse performance or clinical competence, and 13 (32.5%) addressed formal appraisal processes or systems; these domains were not mutually exclusive. Only four studies (10.0%) explicitly examined outcomes directly in relation to an appraisal process or system. Methodological quality varied across the heterogeneous study designs, and direct evidence regarding the effects of formal performance appraisal on subsequent outcomes was limited.

**Conclusion:**

Nurse performance evaluation is characterized by substantial conceptual and methodological heterogeneity. The evidence supports distinguishing performance and competence measurement from formal performance‐appraisal processes and considering multilevel contextual determinants when interpreting measured nurse performance. Direct evidence regarding the effects of formal appraisal systems on subsequent outcomes remains limited. The proposed framework should therefore be regarded as an evidence‐informed conceptual synthesis requiring prospective validation rather than as a validated predictive or causal model.

**Implications for Nursing Management:**

Healthcare managers should distinguish performance or competence measurement from formal performance‐appraisal processes and select appraisal approaches according to their intended purpose, measurement properties, feasibility, and clinical context. Transparent criteria, evaluator preparation, and meaningful feedback may support acceptable appraisal processes; however, further prospective research is needed to establish the effects of specific appraisal approaches on nurse‐ and patient‐level outcomes.

## 1. Introduction

Nurse performance is central to the delivery of effective healthcare and encompasses the knowledge, skills, professional behaviors, and work‐related activities through which nurses fulfill their clinical and organizational roles. Evaluating this performance is therefore relevant to nursing management, professional development, workforce planning, and quality‐improvement activities [1–3]. However, the literature uses several related concepts including performance measurement, competence assessment, performance evaluation, and performance appraisal that are not always clearly distinguished [3–5].

In this review, nurse performance refers to measured work‐related behaviors, activities, or outcomes relevant to nurses’ professional roles. Professional or clinical competence is considered relevant when it is explicitly assessed as a component or proxy of professional performance. Performance measurement refers to the use of instruments, criteria, indicators, or models to quantify or characterize aspects of performance or competence. In contrast, performance appraisal refers to a structured organizational process through which nurses’ work‐related performance, competence, or professional behaviors are evaluated for purposes such as assessment, feedback, professional development, or organizational decision‐making. Performance measurement may therefore contribute to formal appraisal, but the two concepts are not synonymous.

A wide range of approaches has been used to evaluate nursing performance, including self‐report instruments, structured performance and competence measures, supervisor‐based assessment, multisource feedback, competency frameworks, and quantitative or model‐based evaluation approaches [3, 4, 6–9]. The diversity of these approaches reflects differences in the purposes, constructs, clinical contexts, and organizational settings of performance evaluation. It also creates challenges for comparing findings across studies and determining which approaches are appropriate for particular evaluative purposes.

Nurse performance is also examined in relation to a range of individual, professional, organizational, managerial, and work‐environment factors. Characteristics such as professional experience, self‐efficacy, motivation, work engagement, leadership, workload, organizational support, and workplace conditions have been investigated in relation to measured performance or competence [2, 10–15]. These relationships suggest that performance evaluation may need to be interpreted within the broader context in which nursing practice occurs rather than solely as an assessment of individual attributes. However, associations between such factors and measured performance should not be assumed to represent causal relationships.

Formal performance‐appraisal systems add a further organizational dimension to performance evaluation by combining assessment with processes such as managerial review, feedback, appraisal interviews, and professional‐development planning [4, 16–19]. Appraisal may be intended to support professional development and organizational decision‐making, but the extent to which formal appraisal processes themselves produce improvements in nurse performance or broader organizational and patient outcomes remains uncertain. In particular, relationships between appraisal and outcomes such as quality of care or patient safety require direct empirical evaluation rather than inference from studies of nurse performance more generally.

Previous literature has addressed particular instruments, competencies, determinants, or appraisal practices, but these components have often been examined separately or described using overlapping terminology [2–4, 8, 19]. An integrated synthesis that preserves the distinction between measurement of performance or competence, contextual determinants of measured performance, formal appraisal processes, and outcomes directly related to appraisal may therefore provide a clearer representation of the available evidence.

Accordingly, this systematic review aimed to synthesize primary empirical evidence on nurse performance evaluation in clinical healthcare settings and to develop an evidence‐informed conceptual framework integrating the principal components identified across the literature. Specifically, the review addressed the following questions:

1. What tools, instruments, criteria, models, and methods have been used to measure or evaluate nurse performance or clinically relevant professional competence?
2. What individual, professional, organizational, managerial, and work‐environment factors have been examined in relation to measured nurse performance or clinical competence?
3. What formal performance‐appraisal processes and systems have been investigated in nursing practice?
4. What outcomes have been examined directly in relation to formal nurse performance‐appraisal processes or systems?
5. How can these findings be integrated into an evidence‐informed conceptual framework for understanding nurse performance evaluation?

## 2. Methods

### 2.1. Study Design and Reporting Standards

This systematic review was conducted and reported in accordance with the Preferred Reporting Items for Systematic Reviews and Meta‐Analyses (PRISMA) 2020 statement. A structured approach was used for study identification, screening, eligibility assessment, data extraction, methodological appraisal, and evidence synthesis.

The review focused on primary empirical and methodological studies addressing nurse performance appraisal and evaluation, including tools and methods used to measure nurse performance or clinically relevant professional competence, determinants associated with measured nurse performance or competence, formal performance‐appraisal processes and systems, and outcomes examined directly in relation to appraisal processes. Given the conceptual and methodological heterogeneity of the evidence, findings were synthesized qualitatively.

### 2.2. Protocol Registration

This systematic review was not prospectively registered in PROSPERO, and no prospectively registered review protocol was available. The review was conducted and reported in accordance with PRISMA 2020 [20]. The absence of prospective protocol registration is acknowledged as a methodological limitation because it limits independent verification of whether methodological decisions changed during the review process.

### 2.3. Search Strategy

A systematic literature search was conducted across five electronic bibliographic databases: PubMed, Scopus, Web of Science, CINAHL, and Embase. The search covered studies published from January 2010 to November 2025. The final searches were conducted on May 9, 2026, in all five databases.

Database‐specific controlled vocabulary, where applicable, together with free‐text terms, was used to identify studies addressing nurses and performance appraisal or performance evaluation. Search terminology included terms relating to nurses and nursing staff in combination with terms such as “performance appraisal,” “staff appraisal,” “employee performance appraisal,” “performance review,” “annual appraisal,” “performance feedback,” “nurse performance,” “nursing performance,” “nursing performance assessment,” “nurse performance assessment,” “nursing performance measurement,” and “nursing performance indicators.” Search syntax was adapted to the indexing structure and search interface of each database.

The documented database searches retrieved 4527 records before deduplication: 816 from PubMed, 603 from Web of Science, 832 from CINAHL, 825 from Embase, and 1451 from Scopus. The database‐specific strategies reported in Supporting File 1 correspond to the actual final searches conducted on May 9, 2026, and generated these retrieval counts; they were not reconstructed from later exploratory reruns. The complete database‐specific search strategies and retrieval counts are provided in Supporting File 1.

The review considered publications in English, Chinese, Turkish, Persian, and Korean. The language restriction reflected the linguistic and practical resources available to the review team for reliable full‐text assessment. Where necessary, translation was used to support full‐text assessment; specifically, the Korean‐language study included in the review was translated before eligibility assessment and data extraction. Because studies published in other languages may have been missed, potential language bias was considered when interpreting the findings. No additional information sources are reported because reproducible search documentation was retained only for the five bibliographic databases described above.

### 2.4. Eligibility Criteria

Studies were considered eligible according to predefined criteria relating to population, concept, context, study design, publication period, and language.

#### 2.4.1. Population

Studies involving registered nurses working in clinical healthcare settings, including hospitals, primary care, community settings, and other clinical practice environments, were eligible. Studies involving nursing students, other healthcare professionals, or mixed professional populations were excluded when nurse‐specific data could not be separately identified. Mixed nursing and midwifery populations were considered only when the study directly evaluated an appraisal or competence framework applicable to nursing practice and its relevance to nurse performance appraisal could be clearly established.

#### 2.4.2. Concept

Eligible studies were required to address at least one of the following components of nurse performance appraisal or evaluation: (1) formal performance‐appraisal systems, processes, interviews, feedback mechanisms, or implementation approaches; (2) development, validation, or application of instruments, scales, criteria, models, or frameworks explicitly intended to assess nurses’ work performance, professional performance, or clinically relevant competence for evaluative purposes; or (3) individual, professional, organizational, or environmental determinants examined in relation to an explicitly measured nurse‐performance or clinical‐competence outcome.

For the purposes of this review, nurse performance appraisal was defined as a structured process or measurement approach used to evaluate nurses’ work‐related performance, competence, or professional behaviors for assessment, feedback, professional development, or organizational decision‐making. Nurse performance referred to measured work‐related behaviors, competencies, or performance outcomes. Studies assessing professional or clinical competence were eligible only when competence was explicitly evaluated as a component or proxy of professional nursing performance among practicing nurses.

Studies focusing solely on related constructs such as job satisfaction, self‐efficacy, leadership, work environment, education, training, patient satisfaction, or quality of care were not eligible on the basis of those constructs alone. Such studies were eligible only when these factors were directly examined in relation to an explicitly measured nurse performance or clinical competence outcome or were directly associated with a formal performance‐appraisal process or system.

### 2.5. Study Design

Eligible designs included quantitative observational studies, longitudinal studies, qualitative studies, mixed‐methods studies, quasiexperimental and experimental studies, and methodological studies involving instrument development, psychometric validation, competency assessment, or performance‐evaluation model development. Systematic reviews, narrative reviews, scoping reviews, editorials, commentaries, expert opinions, methodological guidelines, conference abstracts without sufficient empirical data, and other nonempirical publications were excluded from the primary synthesis. Relevant review articles identified during the search were retained only as contextual sources for the Introduction or Discussion; they were not counted among the included primary studies, subjected to primary‐study data extraction or methodological appraisal, or incorporated into the primary thematic synthesis.

Context: Studies conducted in clinical healthcare settings across different geographical and healthcare‐system contexts were eligible, provided that the population and concept criteria were satisfied.

### 2.6. Time Frame

Studies published between January 2010 and November 2025 were considered eligible.

Language: Articles published in English, Chinese, Turkish, Persian, or Korean were eligible.

Eligibility assessment was performed independently by two reviewers. Disagreements were resolved through discussion and consensus and, when necessary, consultation with a third reviewer.

Studies were excluded if they involved an ineligible population; did not address nurse performance, clinically relevant competence, or a formal performance‐appraisal process as defined above; did not provide eligible empirical or methodological data; were secondary or nonempirical publications; represented duplicate publications; or were unavailable in full text.

### 2.7. Data Extraction

Data were extracted using a standardized extraction form developed for this review. Extracted information included authorship, year of publication, country and clinical setting, study design, sample characteristics, study objectives, performance‐appraisal or measurement approach, instruments or frameworks used to assess nurse performance or clinically relevant professional competence, determinants examined in relation to measured performance, appraisal‐related outcomes, and key findings relevant to the review questions.

For synthesis purposes, extracted evidence was classified into four overlapping domains: (1) tools and methods for measuring nurse performance or clinically relevant professional competence; (2) individual, professional, organizational, and work‐environment determinants associated with measured nurse performance or competence; (3) formal performance‐appraisal processes and systems; and (4) outcomes examined directly in relation to a performance‐appraisal process or system. Individual studies could contribute to more than one domain when appropriate.

Data extraction was performed independently by two reviewers using the standardized form. Discrepancies were resolved through discussion and consensus and, when necessary, consultation with a third reviewer. The extraction form was pilot‐tested on a subset of included studies and refined before full data extraction. Extracted data were subsequently cross‐checked for completeness and consistency before synthesis.

### 2.8. Quality Appraisal

Methodological quality was assessed using design‐appropriate critical appraisal approaches. Primary empirical studies corresponding to study categories covered by the Mixed Methods Appraisal Tool (MMAT), 2018 version [21], were appraised using the relevant MMAT criteria. The two MMAT screening questions were considered first, followed by the five criteria specific to the applicable study‐design category. Each criterion was recorded individually as “Yes,” “No,” or “Can’t tell,” and no overall numerical MMAT score was calculated.

Studies primarily concerned with instrument development or psychometric evaluation were assessed using measurement‐property criteria appropriate to their methodological purpose rather than being assigned to an MMAT category. Similarly, Delphi, decision‐analysis, performance‐model, and framework‐development studies were evaluated using methodological criteria appropriate to their respective designs.

Two reviewers independently conducted the methodological appraisal. Disagreements were resolved through discussion and consensus and, when necessary, consultation with a third reviewer. Quality‐appraisal findings were used to contextualize interpretation of the synthesis rather than as a basis for excluding studies. No post hoc sensitivity analysis based on an aggregate methodological‐quality score was performed. No formal assessment of reporting bias or certainty of the overall body of evidence was undertaken. Detailed study‐level judgments and methodological justifications are provided in Supporting File 3.

### 2.9. Data Synthesis

Given the heterogeneity of study designs, appraisal and measurement approaches, determinants, and reported outcomes, a meta‐analysis was not considered appropriate. Findings were therefore synthesized qualitatively using a thematic synthesis approach informed by Thomas and Harden [22].

The synthesis involved three stages: (1) coding of findings relevant to the review questions; (2) organization of related codes into descriptive categories and themes; and (3) interpretive integration of the identified themes across studies. Coding and synthesis focused on four overlapping evidence domains: tools and methods for measuring nurse performance or clinically relevant professional competence; determinants associated with measured nurse performance or competence; formal performance‐appraisal processes and systems; and outcomes examined directly in relation to appraisal processes.

Initial coding was conducted independently by two reviewers using NVivo version 12 to facilitate data organization and management. Codes were compared and refined through iterative discussion. Individual studies were permitted to contribute to more than one evidence domain when their findings addressed multiple components of the review scope.

In the interpretive stage, relationships across the identified domains were examined to develop an evidence‐informed conceptual synthesis of nurse performance evaluation, its multilevel determinants, formal appraisal processes, and appraisal‐related outcomes. Because the included evidence was heterogeneous and predominantly observational or methodological, the resulting framework was interpreted as a conceptual organization of the available evidence rather than as a validated predictive or causal model. Relationships identified across studies were therefore not interpreted as causal or mediational unless they had been formally tested within the original study.

Regular discussions between the reviewers were used to resolve coding differences, refine the thematic structure, and maintain consistency throughout the synthesis.

### 2.10. Ethical Considerations

Ethical approval was not required because this systematic review synthesized data from previously published studies and did not involve direct participation of human participants or access to identifiable personal data. The review was conducted and reported in accordance with established systematic‐review reporting principles, including PRISMA 2020.

## 3. Results

### 3.1. Study Selection

The final searches of the five bibliographic databases retrieved 4527 records before deduplication, comprising 816 records from PubMed, 603 from Web of Science, 832 from CINAHL, 825 from Embase, and 1451 from Scopus. The exact historical number of duplicate records removed and the exact number of records entering title and abstract screening could not be reliably reconstructed from the retained search documentation and were therefore not retrospectively estimated.

Following title‐and‐abstract screening, 328 reports were sought for full‐text retrieval. Twelve reports could not be retrieved, leaving 316 reports assessed for eligibility. Of these, 276 were excluded: 122 were outside the predefined conceptual scope, 76 did not provide eligible empirical data, 47 involved ineligible or non‐nursing populations, 30 were reviews, editorials, or commentaries, and one was a duplicate publication identified during the final eligibility audit.

Accordingly, 40 primary empirical studies met the eligibility criteria and were included in the qualitative synthesis. Two relevant review articles identified during the search were retained only as contextual sources and were not counted among the included primary studies or incorporated into the primary thematic synthesis.

The study‐selection process and reasons for full‐text exclusion are presented in Figure 1 and Supporting File 2.

**FIGURE 1:**
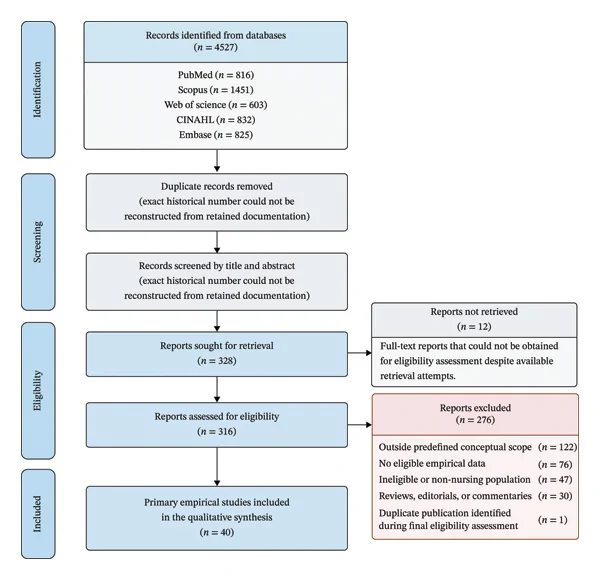
PRISMA flow diagram of study identification, screening, eligibility assessment, and inclusion. Note. Database‐specific retrieval counts were verified from the retained search documentation. The exact historical numbers of duplicate records removed and records entering title/abstract screening could not be reliably reconstructed and were therefore not retrospectively estimated.

### 3.2. Characteristics of the Included Studies

The 40 primary studies included in the qualitative synthesis were published between 2010 and 2025 and represented diverse geographical and healthcare contexts. Studies from Türkiye were most frequent (*n* = 5, 12.5%), followed by Taiwan (*n* = 4, 10.0%) and Indonesia (*n* = 4, 10.0%). Studies were also conducted across East and South Asia, the Middle East, Europe, Africa, and South America, indicating broad geographical representation alongside substantial contextual heterogeneity.

Cross‐sectional designs, including comparative, multilevel, and descriptive‐comparative cross‐sectional variants, were the most common (*n* = 21, 52.5%). The remaining evidence comprised instrument‐development and validation studies, descriptive studies, longitudinal and qualitative research, quasiexperimental and randomized studies, Delphi and decision‐analysis studies, and performance‐model or framework‐development studies. Overall, observational and methodological studies predominated, whereas longitudinal and intervention‐based evidence remained limited.

Conceptually, the evidence addressed four overlapping domains aligned with the scope of this review: (1) tools and methods for performance or clinically relevant competence measurement; (2) individual, professional, organizational, and work‐environment determinants of measured nurse performance or competence; (3) formal performance‐appraisal processes and systems; and (4) outcomes examined directly in relation to performance‐appraisal processes. Individual studies could contribute to more than one domain.

Table 1 presents representative studies illustrating the temporal and methodological diversity of the included evidence. Complete characteristics of all 40 primary studies, including country, study design, setting, appraisal or performance measure, review domain, main focus, and key findings, are provided in Supporting Table S1.

**TABLE 1:** Representative characteristics and methodological developments across the included primary studies.

Period	Representative study	Country	Study design/approach	Main focus
2010–2015	Lin et al. [8]	Taiwan	Instrument development/validation	Measurement of nursing competence
	Salanova et al. [13]	Portugal	Observational	Organizational and work‐environment factors associated with nurse performance
	Awases et al. [23]	Namibia	Cross‐sectional	Factors associated with nurses’ performance
	Nikpeyma et al. [19]	Iran	Qualitative	Nurses’ experiences and perceptions of performance appraisal
	Fujino et al. [24]	Japan	Cross‐sectional	Work‐related factors associated with nursing performance

2016–2020	Lee [25]	South Korea	Instrument development/validation	Measurement of nursing performance
	Youssif et al. [26]	Egypt	Observational	Performance appraisal, job satisfaction, and empowerment
	Holanda et al. [7]	Brazil	Instrument validation	Assessment of professional nursing competence
	Chen et al. [12]	Taiwan	Longitudinal	Factors associated with nurses’ work performance
	Soares et al. [3]	Brazil	Descriptive study	Structured assessment of nursing professional performance
	Bigdeli et al. [17]	Iran	Intervention‐based study	Structured appraisal interview and nurse performance

2021–2025	Cheng et al. [6]	Taiwan	Decision‐analysis/model development	Quantitative evaluation of nursing competence/performance
	Sullivan et al. [27]	Qatar	Framework development/pilot	Appraisal and competence framework
	Yang [28]	China	Performance‐model development	Quantitative evaluation of nursing performance
	Gedük et al. [29]	Türkiye	Instrument/implementation study	Structured nursing performance evaluation
	Jaber et al. [18]	Saudi Arabia	Cross‐sectional descriptive comparative study	Perceived efficacy of the performance‐appraisal system and work‐related outcomes

*Note:* Representative studies were selected to illustrate the temporal, geographical, and methodological diversity of the included evidence. The table is not intended to provide an exhaustive list of the 40 primary studies. Evidence domains are not mutually exclusive, and individual studies may contribute to more than one domain. Complete characteristics and domain classifications for all 40 included primary studies are provided in Supporting Table S1.

### 3.3. Performance Appraisal and Measurement Approaches

Sixteen of the 40 included primary studies (40.0%) contributed to the tools and measurement domain. The approaches identified across these studies were heterogeneous and reflected different purposes, including measurement of overall nursing performance, assessment of clinical or professional competence, structured performance evaluation, multisource feedback, development of appraisal or competence frameworks, and quantitative or multicriteria performance evaluation.

Self‐report questionnaires and scales were used in several studies to assess nurses’ work‐related or professional performance. Other studies developed or applied structured instruments designed to evaluate clinical or professional competence as a component or proxy of nursing performance. Examples included competence‐oriented instruments and frameworks [6–8, 30]. Structured nursing‐performance instruments were also developed or evaluated, including those [3, 25, 29].

Several studies used approaches extending beyond conventional self‐report measurement. Model‐based approaches were also identified; for example, Cheng et al. [6] and Osman et al. [9] applied quantitative analytical approaches to evaluate nurse performance or relative performance.

Crossley [4] examined multisource feedback, whereas Sullivan et al. [27]; Nuryanti et al. [31]; and Nuritasari et al. [32] addressed structured appraisal instruments or competence frameworks. Consensus‐based and analytical approaches were also represented. Li et al. [30] used an expert‐consensus approach for competency development, while Cheng et al. [6, 9, 28] applied quantitative or multicriteria approaches to performance or competence evaluation. Context‐ or unit‐specific approaches were represented by studies such as [5, 33, 34].

Importantly, these approaches should not be interpreted as equivalent forms of formal performance appraisal. Some studies primarily addressed measurement of nurse performance or clinically relevant professional competence, whereas others examined structured appraisal processes or systems. Accordingly, the review distinguishes performance and competence measurement from formal performance‐appraisal processes while recognizing that individual studies may contribute to both domains. The principal measurement and appraisal approaches identified across the included evidence are summarized in Table 2.

**TABLE 2:** Principal performance appraisal and measurement approaches identified across the included studies.

Measurement/appraisal approach	Description and purpose	Representative included studies
Self‐report performance measurement	Structured questionnaires or scales completed by nurses to assess dimensions of their own work‐related or professional performance.	Chen et al. [35]; Asiamah et al. [1]; Cho and Han [36]; Bhatti et al. [11]
Clinical/professional competence instruments	Structured instruments designed to assess clinical, professional, or role‐specific nursing competence as an evaluative component or proxy of professional performance.	Lin et al. [8]; Holanda et al. [7]; Cheng et al. [6]; Li et al. [30]
Structured nursing performance instruments	Instruments developed or validated specifically to measure multidimensional nursing performance or support structured performance evaluation.	Lee [25]; Soares et al. [3]; Gedük et al. [29]
Multisource feedback	Performance assessment incorporating evaluations from multiple sources rather than relying exclusively on self‐assessment.	Crossley [4]
Formal appraisal and competence frameworks	Structured systems or frameworks integrating performance evaluation, competence assessment, appraisal processes, feedback, and/or professional development.	Sullivan et al. [27]; Nuritasari et al. [32]
Consensus‐based competency development	Expert‐consensus approaches used to identify, structure, or prioritize competency and performance indicators for evaluative purposes.	Li et al. [30]
Multicriteria and quantitative performance‐evaluation models	Analytical approaches integrating multiple performance criteria or indicators to support multidimensional or relative performance evaluation.	Cheng et al. [6]; Osman et al. [9]; Yang [28]
Context‐ or unit‐specific appraisal tools	Performance‐assessment instruments, criteria, or models developed for particular nursing roles, units, clinical contexts, or organizational settings.	Kahya and Oral [33]; Kahya and Oral [5]; Chen et al. [34]

*Note:* Categories represent the principal measurement and appraisal approaches identified across the included evidence and are not mutually exclusive; individual studies may contribute to more than one category. The table distinguishes measurement of nurse performance or clinically relevant professional competence from formal performance‐appraisal processes. Sixteen of the 40 included studies (40.0%) contributed to the broader tools/measurement domain. Complete study‐level classifications are provided in Supporting Table S1.

### 3.4. Determinants of Measured Nurse Performance or Clinical Competence

Nineteen of the 40 included primary studies (47.5%) examined determinants associated with explicitly measured nurse performance or clinically relevant professional competence. The identified determinants were heterogeneous and operated across multiple, overlapping levels. For synthesis, they were organized into three broad categories: individual and professional factors, organizational and managerial factors, and work‐environment and relational factors (Table 3).

**TABLE 3:** Principal determinants of measured nurse performance or clinical competence identified across the included studies.

Determinant category	Factors represented in the included evidence	Representative included studies
Individual and professional factors	Self‐efficacy, motivation, work engagement, emotional intelligence, education and training, professional experience, and competency‐related characteristics.	Chen et al. [35]; Sharma and Dhar [14]; Tran et al. [15]; Sarıköse and Göktepe [2]; Awases et al. [23]; Al‐Makhaita et al. [37]; Chairina et al. [38]; Fujino et al. [24]; Kim et al. [39]; Bhatti et al. [11]; Salanova et al. [13]
Organizational and managerial factors	Leadership and managerial support, workload and staffing conditions, job resources, and broader organizational conditions associated with measured nurse performance or competence.	Asiamah et al. [1]; Cho and Han [36]; Widyana and Handiyani [40]; Top [41]; Al‐Hawary and Banat [10]; Kamati et al. [42]
Work‐environment and relational factors	Characteristics of the work environment, organizational climate, workplace relationships, and contextual resources associated with measured nurse performance or competence.	Sharma and Dhar [14]; Sarıköse and Göktepe [2]; Cho and Han [36]; Geyer et al. [43]; Shen et al. [44]; Salanova et al. [13]

*Note:* Determinant categories are overlapping and are not mutually exclusive; individual studies may have examined factors across more than one level. Only studies in which determinants were examined in relation to an explicitly measured nurse‐performance or clinical‐competence outcome were classified within this domain. Nineteen of the 40 included studies (47.5%) contributed to the determinant domain. Complete study‐level classifications are provided in Supporting Table S1.

Individual and professional factors included self‐efficacy, motivation, work engagement, emotional intelligence, education and training, professional experience, and competency‐related characteristics. These factors were examined in studies including [2, 10, 11, 13–15, 23, 24, 35, 37–39]. The direction and magnitude of reported relationships varied across studies and contexts; therefore, these factors should not be interpreted as having uniform effects on nurse performance.

Organizational and managerial factors included leadership and managerial support, workload and staffing conditions, job resources, and broader organizational conditions associated with measured performance or competence. Relevant evidence was reported in studies such as [1, 10, 36, 40–42]. Collectively, these studies indicate that measured nurse performance is situated within an organizational context rather than being attributable solely to individual characteristics.

Work‐environment and relational factors included characteristics of the work environment, organizational climate, workplace relationships, and contextual resources. Studies contributing to this category included [2, 13, 14, 36, 41, 44].

Some studies contributed to more than one determinant category, reflecting the conceptual overlap between individual, organizational, and work‐environment influences.

Overall, the determinant evidence supports a multilevel interpretation of measured nurse performance and competence. However, because much of this evidence was observational, the identified relationships should primarily be interpreted as associations rather than established causal effects. The principal determinant categories and representative studies are summarized in Table 3.

### 3.5. Performance and Evaluation Domains

The constructs assessed across the included studies reflected substantial conceptual diversity. Six broad performance and evaluation domains were identified: overall or job‐related nursing performance; clinical and professional competence; task and contextual performance; appraisal effectiveness and fairness; professional and caring behaviors; and efficiency or relative performance (Table 4). These domains were not mutually exclusive, and individual studies could address more than one aspect of performance or evaluation.

**TABLE 4:** Performance and evaluation domains represented in the included evidence.

Performance/evaluation domain	Description	Representative included studies
Overall or job‐related nursing performance	Global or multidimensional assessment of nurses’ work‐related performance, including performance measures used in studies examining individual, professional, or contextual determinants.	Chen et al. [12]; Sharma and Dhar [14]; Tran et al. [15]; Awases et al. [23]; Chairina et al. [38]
Clinical and professional competence	Assessment of clinical knowledge, skills, professional capabilities, or role‐specific competence as an evaluative component or proxy of nursing performance.	Lin et al. [8]; Holanda et al. [7]; Cheng et al. [6]; Li et al. [30]; Sullivan et al. [27]
Task and contextual performance	Evaluation of core task execution and contextual work behaviors contributing to effective nursing performance.	Fujino et al. [24]; Bhatti et al. [11]
Appraisal effectiveness and fairness	Evaluation of nurses’ perceptions of appraisal‐system effectiveness, fairness, usefulness, or quality.	Youssif et al. [26]; Ali et al. [16]; Jaber et al. [18]
Professional and caring behaviors	Assessment of professional behaviors, role performance, interpersonal practice, or caring‐related components incorporated within broader nursing‐performance measures.	Soares et al. [3]; Lee [25]
Efficiency and relative performance	Quantitative or model‐based evaluation of nursing performance using multidimensional indicators, efficiency measures, or relative performance criteria.	Osman et al. [9]; Yang [28]; Cheng et al. [6]

*Note:* Performance and evaluation domains were derived from constructs explicitly measured or evaluated in the included studies. Domains are overlapping and are not mutually exclusive, and individual studies may contribute to more than one domain. Outcomes such as job satisfaction, patient satisfaction, or quality of care were not classified as nurse‐performance domains unless they formed part of an explicitly defined and measured nurse‐performance construct.

Overall or job‐related nursing performance was examined as a global or multidimensional construct in several studies, particularly those investigating factors associated with nurses’ measured work performance. Representative studies included [12, 14, 15, 23, 38].

Clinical and professional competence represented another prominent evaluative domain. Studies such as [6–8, 27, 30], assessed clinical knowledge, professional skills, role‐specific capabilities, or broader competence constructs. Within this review, competence measures were considered relevant only when they were explicitly used as an evaluative component or proxy of professional nursing performance among practicing nurses.

Other studies addressed more specific dimensions of performance and evaluation. Task and contextual performance concerned core task execution and broader work behaviors contributing to effective nursing performance, as represented in studies such as [11, 24]. Professional and caring behaviors were represented within structured nursing‐performance instruments incorporating professional, interpersonal, or caring‐related components, as reported in studies such as [3, 25]. In addition, quantitative and model‐based studies examined efficiency or relative performance using multiple criteria or performance indicators, as demonstrated by [6, 9, 28].

A conceptually distinct domain concerned appraisal effectiveness and fairness. Studies by [16, 26, 44] examined perceptions of formal appraisal processes or systems, including their effectiveness, fairness, usefulness, or related appraisal characteristics. These constructs were distinguished from direct measures of nurses’ work performance or clinical competence.

Importantly, broader outcomes such as job satisfaction, empowerment, patient satisfaction, quality of care, and patient safety were not treated as nurse‐performance domains merely because they appeared in studies related to nursing practice. Outcomes were considered appraisal‐related only when they were examined directly in relation to a formal performance‐appraisal process or system. This distinction was maintained throughout the synthesis to avoid conflating nurse‐performance measurement with downstream organizational, professional, or patient‐level outcomes.

### 3.6. Appraisal‐Related Outcomes

Evidence regarding outcomes directly associated with formal performance‐appraisal processes or systems was limited. Only four of the 40 included primary studies (10.0%) explicitly examined outcomes in direct relation to an appraisal process or system (Table 5).

**TABLE 5:** Outcomes examined directly in relation to performance appraisal processes or systems.

Study	Appraisal process/system examined	Appraisal‐related outcome	Main finding
Youssif et al. [26]	Nurses’ perceptions of the performance‐appraisal process	Job satisfaction and empowerment	Perceptions of performance appraisal were associated with nurses’ job satisfaction and empowerment.
Bigdeli et al. [17]	Structured performance‐appraisal interview	Nurse performance	Nurses receiving the structured appraisal interview demonstrated improved measured performance compared with routine evaluation.
Ali et al. [16]	Training of nurse managers in performance appraisal	Perceived appraisal fairness and collaboration	The intervention was associated with favorable changes in nurses’ perceptions of appraisal fairness and collaboration.
Jaber et al. [18]	Perceived efficacy of the performance‐appraisal system	Work‐related outcomes	Perceptions of appraisal‐system efficacy varied across professional roles and were associated with work‐related outcomes.

*Note:* Only studies that examined outcomes directly in relation to a formal performance‐appraisal process or system were included in this table. Four of the 40 included primary studies (10.0%) met this criterion. Because these studies differed substantially in design, appraisal approach, comparator, and outcome definition, their findings should not be interpreted as evidence of a uniform or causal effect of performance appraisal.

Youssif et al. [26] examined nurses’ perceptions of the performance‐appraisal process and reported associations with job satisfaction and empowerment. Bigdeli et al. [17] evaluated a structured performance‐appraisal interview and reported improved measured nurse performance among nurses receiving the structured appraisal approach compared with routine evaluation. Ali et al. [16] examined training of nurse managers in performance appraisal and reported favorable changes in nurses’ perceptions of appraisal fairness and collaboration. Jaber et al. [18] examined the perceived efficacy of the performance‐appraisal system and reported relationships between appraisal‐system perceptions and work‐related outcomes.

These findings should not be interpreted as evidence of a uniform effect of performance appraisal. The four studies differed in study design, appraisal approach, comparator, and outcome definition, and only a limited subset of the evidence involved intervention‐based designs. Consequently, causal conclusions regarding the effects of formal performance appraisal cannot be drawn from the evidence base as a whole.

Furthermore, studies of education, professional development, leadership, work environment, or other organizational factors were not interpreted as evidence of appraisal‐system effectiveness unless the intervention or exposure was explicitly linked to a formal performance‐appraisal process or system. Similarly, patient satisfaction, quality of care, and patient safety were not considered established outcomes of performance appraisal in the absence of direct evidence linking them to an appraisal process.

The appraisal processes, outcomes examined, and principal findings of the four directly relevant studies are summarized in Table 5.

### 3.7. Methodological Quality of Included Studies

Methodological quality varied across the included studies, reflecting the heterogeneity of study designs and methodological purposes. Studies corresponding to established MMAT design categories were assessed using the relevant MMAT 2018 criteria [21], whereas studies primarily involving instrument development or psychometric evaluation, Delphi consensus, decision analysis, performance‐model development, or framework development were evaluated using design‐appropriate appraisal criteria.

Among the observational studies, recurrent methodological uncertainties concerned the representativeness of study populations, potential nonresponse bias, completeness of outcome reporting, and, where relevant, the extent to which potential confounding was adequately addressed. The qualitative evidence demonstrated overall coherence between the research questions, data‐collection approaches, and analyses, although reporting relevant to researcher influence and reflexivity was limited. For the randomized and quasiexperimental evidence, the available reporting did not permit all relevant risk‐of‐bias domains to be judged positively.

Methodological studies generally demonstrated a clear rationale for instrument, competency‐framework, or performance‐model development. However, the extent of psychometric testing, reliability assessment, measurement‐error evaluation, external validation, and assessment of model robustness varied across studies or was incompletely reported. Delphi, decision‐analysis, and model‐ or framework‐development studies were therefore interpreted according to methodological criteria appropriate to their respective designs rather than being assigned to an inappropriate MMAT category.

Overall, the methodological appraisal did not support uniformly positive quality judgments across the evidence base. These limitations were considered when interpreting the synthesis, particularly given the predominance of observational and methodological evidence and the limited amount of intervention‐based evidence. No aggregate methodological‐quality score was calculated, and no post hoc sensitivity analysis based on such a score was performed. No formal assessment of reporting bias or certainty of the overall body of evidence was undertaken. Detailed study‐level appraisal judgments and methodological justifications are provided in Supporting File 3.

### 3.8. Evidence‐Informed Conceptual Framework

An evidence‐informed conceptual framework was developed to integrate the principal domains identified across the included studies (Figure 2). The framework distinguishes four overlapping components of the evidence base: Approaches used to measure nurse performance or clinically relevant professional competence; individual, professional, organizational, and work‐environment determinants associated with measured performance or competence; formal performance‐appraisal processes and systems; and outcomes examined directly in relation to appraisal processes.

**FIGURE 2:**
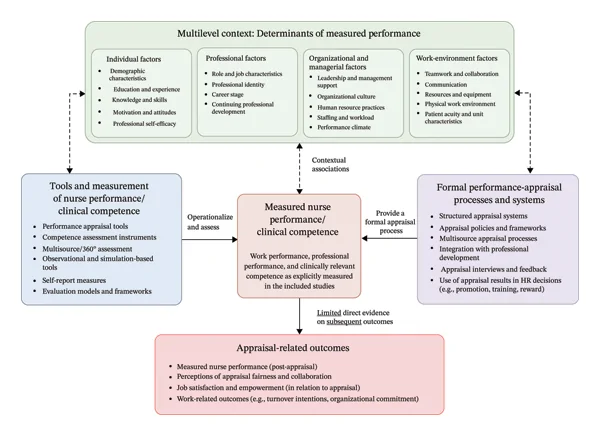
Evidence‐informed conceptual framework of nurse performance evaluation, its determinants, appraisal processes, and appraisal‐related outcomes. The framework synthesizes the principal domains identified across the included studies. Connections represent conceptual relationships observed across the evidence base and should not be interpreted as established causal pathways. Evidence concerning outcomes directly attributable to formal performance‐appraisal processes was limited. Note. The framework synthesizes the principal domains identified across the included studies. Solid connectors indicate components directly linked within appraisal or measurement processes, whereas dashed connectors indicate contextual associations represented in the included evidence. Connections represent evidence‐informed conceptual relationships and should not be interpreted as established causal or mediational pathways unless such relationships were formally tested in the original studies. Evidence domains overlap, and individual studies may contribute to more than one component. Evidence concerning outcomes directly attributable to formal performance‐appraisal processes was limited.

The synthesis suggests that measured nurse performance is situated within a multilevel context in which individual and professional characteristics coexist with organizational, managerial, and work‐environment conditions, as reflected across studies examining individual, organizational, and contextual determinants of performance [2, 11, 13–15, 36, 38, 41]. Formal performance‐appraisal processes represent a related but conceptually distinct component, encompassing structured appraisal systems, appraisal interviews, feedback processes, multisource approaches, and integrated appraisal or competence frameworks, as represented by studies including [4, 16, 17, 19, 26, 27, 29]. Measurement approaches provide the means through which performance or clinically relevant competence is operationalized and evaluated across these contexts.

Evidence directly linking formal appraisal processes to subsequent outcomes was comparatively limited. The identified appraisal‐related outcomes included changes in measured nurse performance, perceptions of appraisal fairness and collaboration, job satisfaction and empowerment in relation to appraisal perceptions, and work‐related outcomes associated with perceived appraisal‐system efficacy. Given the heterogeneity and predominantly observational or methodological nature of the evidence base, these relationships should be interpreted as reported associations or study‐specific findings rather than as established causal pathways.

Accordingly, Figure 2 should be interpreted as an evidence‐informed conceptual synthesis rather than a validated predictive or causal model. The framework is intended to organize the principal constructs identified in the review and to highlight potential relationships requiring further prospective and intervention‐based investigation. It does not imply that all pathways have been empirically tested or that the direction, magnitude, or relative importance of relationships is consistent across healthcare settings.

## 4. Discussion

### 4.1. Principal Findings

This systematic review synthesized evidence from 40 primary empirical studies published between 2010 and 2025 examining nurse performance evaluation across diverse healthcare and geographical contexts. The evidence was conceptually and methodologically heterogeneous and was organized into four overlapping domains: (1) tools and methods for measuring nurse performance or clinically relevant professional competence; (2) individual, professional, organizational, and work‐environment determinants associated with measured performance or competence; (3) formal performance‐appraisal processes and systems; and (4) outcomes examined directly in relation to formal appraisal processes.

A key finding of this review is the distinction between performance or competence measurement and formal performance appraisal. Although these concepts were sometimes treated interchangeably in the literature, they represent related but distinct components of performance evaluation. Measurement studies primarily focused on how nurse performance or clinically relevant competence was operationalized and assessed, whereas formal appraisal studies addressed structured organizational processes involving evaluation, feedback, appraisal interviews, implementation approaches, or appraisal frameworks. Recognizing this distinction provides a more precise interpretation of the heterogeneous evidence base.

The review also identified substantial diversity in approaches to performance and competence measurement. These included self‐report measures, clinical or professional competence instruments, structured nursing‐performance instruments, multisource feedback, formal appraisal and competence frameworks, consensus‐based competency development, and quantitative or multicriteria evaluation models [4, 6–9, 30]. This diversity reflects differences in the purposes and contexts of nurse performance evaluation and limits direct comparison across studies.

Determinants of measured nurse performance or competence were reported across multiple levels. Individual and professional characteristics were examined alongside organizational and managerial conditions and work‐environment or relational factors. Taken together, these findings support a multilevel interpretation of nurse performance in which measured performance is situated within both professional and organizational contexts. However, because much of this evidence was observational, the reported relationships should primarily be interpreted as associations rather than causal effects.

Evidence concerning outcomes directly related to formal performance‐appraisal processes was comparatively limited. Only four of the 40 included studies explicitly examined outcomes in direct relation to an appraisal process or system [16–18, 26], and these studies differed in design, appraisal approach, and outcome definition. Reported outcomes included measured nurse performance, appraisal fairness and collaboration, job satisfaction and empowerment in relation to appraisal perceptions, and other work‐related outcomes. Consequently, the available evidence does not support a general conclusion that formal performance appraisal consistently improves nurse‐, organizational‐, or patient‐level outcomes.

Overall, the findings indicate that nurse performance evaluation is best understood as a multidimensional field encompassing measurement, contextual determinants, formal appraisal processes, and a comparatively limited body of evidence on appraisal‐related outcomes. The evidence‐informed framework developed in this review integrates these components while maintaining their conceptual distinctions. It should therefore be interpreted as an organizing synthesis of the available evidence rather than as a validated causal or predictive model.

### 4.2. Methodological Considerations

The methodological diversity of the included studies is an important consideration when interpreting the findings of this review. The evidence base comprised observational, qualitative, experimental, and quasiexperimental, instrument‐development and validation, Delphi, decision‐analysis, performance‐model, and framework‐development studies. This diversity enabled the review to capture multiple dimensions of nurse performance evaluation but also limited direct comparison across studies and precluded meaningful quantitative pooling.

Observational and methodological studies constituted a substantial proportion of the evidence base, whereas prospective and intervention‐based studies were comparatively limited. Among observational studies, uncertainties related to population representativeness, nonresponse, outcome reporting, and control of potential confounding reduce the extent to which reported associations can be interpreted causally. Similarly, the methodological studies varied in the extent of psychometric testing, reliability assessment, measurement‐error evaluation, external validation, and assessment of model robustness.

These methodological characteristics are particularly relevant to the interpretation of the determinant evidence. Although multiple individuals, professional, organizational, managerial, and work‐environment factors were associated with measured nurse performance or competence, the predominance of observational designs means that temporal direction and causality generally cannot be established. Associations identified across studies should therefore not be interpreted as evidence that modification of a particular determinant will necessarily produce corresponding changes in nurse performance.

Similar caution is warranted when interpreting evidence concerning formal performance appraisal. The relatively small number of studies directly examining appraisal‐related outcomes, together with differences in appraisal processes, study designs, comparators, and outcome definitions, limits conclusions regarding the effectiveness of specific appraisal approaches. In particular, the current evidence base does not permit robust conclusions regarding downstream effects of formal appraisal on patient safety, quality of care, or other patient‐level outcomes.

The use of design‐appropriate methodological appraisal in this review was intended to reflect these differences rather than applying a single appraisal framework indiscriminately across heterogeneous study designs. MMAT 2018 [21] was used for applicable study categories, whereas studies primarily concerned with measurement properties, Delphi consensus, decision analysis, model development, or framework development were considered using criteria appropriate to their methodological purpose. The resulting appraisal identified both methodological strengths and uncertainties across the evidence base and did not support uniformly positive quality judgments. These considerations informed the interpretation of the synthesis rather than being used to generate an aggregate quality score or to exclude studies solely on the basis of methodological appraisal.

### 4.3. Interpretation of Performance Appraisal and Measurement Approaches

The included studies demonstrated substantial diversity in how nurse performance and clinically relevant professional competence were conceptualized and measured. Approaches ranged from self‐report questionnaires and structured performance instruments to competence measures, multisource feedback, appraisal and competence frameworks, consensus‐based approaches, and quantitative or multicriteria evaluation models. Several studies focused on the development or validation of structured instruments for assessing nursing performance or competence, including [7, 8, 25], and [3]. Competency‐oriented measurement approaches were also identified; Li et al. [30] used a Delphi‐based approach to develop a competency evaluation index for nurses. Model‐based approaches were represented by studies such as Osman et al. [9] and Cheng et al. [6], which applied quantitative analytical approaches to evaluate nurse performance or relative performance. This heterogeneity reflects the different purposes for which performance information is generated, including measurement, professional evaluation, feedback, competency assessment, and organizational decision‐making.

An important implication of this diversity is that performance measurement should not be treated as synonymous with formal performance appraisal. A measurement instrument may quantify selected dimensions of nurse performance or competence without constituting an organizational appraisal process. Conversely, a formal appraisal system may incorporate several sources of information, feedback processes, managerial judgments, or professional‐development components in addition to standardized measurement. Multisource feedback and structured appraisal approaches were represented, for example, by [4, 27, 31, 32]. Maintaining this distinction is important when comparing studies and interpreting the evidence on appraisal effectiveness.

The reviewed evidence also indicates that no single measurement approach captures all dimensions of nursing performance. Self‐report measures can provide information about nurses’ perceptions of their own performance but may be influenced by response and self‐assessment biases. Structured instruments and competence measures can provide greater standardization, although their usefulness depends on the relevance of the measured constructs, measurement properties, and the context in which they are applied. Multisource and model‐based approaches may broaden the information considered during evaluation, but the included evidence does not establish their general superiority over other approaches.

Context also appears important when selecting and interpreting performance measures. Instruments developed for particular clinical roles, units, or healthcare systems may not retain the same relevance or measurement properties when transferred to different settings without appropriate evaluation. Similarly, competence frameworks and quantitative models may emphasize different dimensions of professional practice according to their intended purpose and development process. These considerations support attention to construct definition, measurement properties, intended use, feasibility, and contextual applicability when interpreting or implementing performance‐evaluation approaches.

Overall, the evidence supports methodological pluralism in nurse performance evaluation but does not establish a universally preferred appraisal or measurement method. Future comparative studies should examine the validity, reliability, feasibility, acceptability, and practical consequences of different approaches within clearly defined appraisal contexts rather than assuming that greater methodological complexity or the use of multiple information sources necessarily produces better evaluation.

### 4.4. Interpretation of Determinants of Nurse Performance

The findings indicate that measured nurse performance and clinically relevant professional competence are associated with factors operating at multiple and overlapping levels. Nineteen of the 40 included studies contributed to the determinant domain, with relevant factors broadly categorized as individual and professional, organizational and managerial, and work‐environment and relational determinants. This pattern suggests that nurse performance should not be interpreted solely as an individual attribute but as a construct situated within broader professional and organizational contexts.

At the individual and professional level, the included studies examined factors such as self‐efficacy, motivation, work engagement, emotional intelligence, education and training, professional experience, and competency‐related characteristics [2, 10–15, 23, 24, 37–39]. These findings suggest that personal and professional resources may be relevant to how nurses perform within clinical roles. However, the relationships were not uniform across studies, and differences in populations, settings, measurement approaches, and study designs limit direct comparison of the relative importance of individual determinants.

At the organizational and managerial level, leadership and managerial support, workload and staffing conditions, job resources, and broader organizational characteristics were examined in relation to measured nurse performance or competence [1, 10, 36, 40–42]. These findings highlight the importance of considering the organizational conditions within which performance is enacted and evaluated. Performance appraisal that focuses exclusively on individual scores without consideration of relevant organizational conditions may therefore provide an incomplete representation of the context surrounding measured performance.

Work‐environment and relational factors, including organizational climate, workplace relationships, leadership‐related conditions, and contextual resources, further illustrate the contextual nature of nurse performance [2, 13, 14, 36, 43, 44]. These factors may overlap with organizational and managerial determinants, reflecting the interconnected nature of clinical work environments. The overlap observed across determinant categories also indicates that rigid separation of individual and contextual influences may not adequately represent the complexity of nursing practice.

Nevertheless, the determinant evidence should be interpreted cautiously. Much of it was derived from cross‐sectional or other observational designs, and the identified relationships generally represent associations rather than demonstrated causal pathways. Although some studies examined mediational relationships, such findings should be interpreted within the specific designs and contexts in which they were tested and should not be generalized as established causal mechanisms across the evidence base. The current evidence therefore does not establish that modifying any single determinant will necessarily improve nurse performance, nor does it establish a consistent hierarchy of determinants across healthcare settings.

From an appraisal perspective, these findings support contextual interpretation of measured performance rather than attribution of performance exclusively to the individual nurse. Future longitudinal and intervention‐based research is needed to clarify temporal relationships among individual, professional, organizational, and work‐environment factors and to determine whether modification of specific contextual factors produces measurable changes in nurse performance or competence.

### 4.5. Outcomes of Nurse Performance Appraisal

Evidence directly addressing outcomes associated with formal performance‐appraisal processes or systems was limited. Only four of the 40 included primary studies explicitly examined outcomes in direct relation to an appraisal process or system, and the outcomes, appraisal approaches, and study designs differed substantially across these studies. This limited evidence base does not support broad conclusions regarding the overall effectiveness of nurse performance appraisal.

The available studies nevertheless illustrate several types of appraisal‐related outcomes. Youssif et al. [26] reported associations between nurses’ perceptions of performance appraisal, job satisfaction, and empowerment. Bigdeli et al. [17] reported improved measured nurse performance following a structured appraisal interview compared with routine evaluation. Ali et al. [16] reported favorable changes in perceived appraisal fairness and collaboration following training of nurse managers in performance appraisal. Jaber et al. [18] identified relationships between perceived appraisal‐system efficacy and work‐related outcomes. Together, these findings suggest that formal appraisal processes may be associated with outcomes at the performance, perceptual, and work‐related levels, but the small and heterogeneous evidence base precludes conclusions about consistent effects.

The distinction between association and intervention effect is particularly important. Findings from observational studies concerning perceptions of appraisal systems cannot establish that the appraisal process caused the associated outcomes. Although the intervention‐based studies provide more direct evidence regarding specific appraisal approaches, their findings should be interpreted within their individual study designs, settings, and outcome measures rather than generalized to performance‐appraisal systems as a whole.

Importantly, the present review did not identify sufficient direct evidence to conclude that formal nurse performance appraisal improves patient safety, quality of care, or other patient‐level outcomes. Such outcomes may be theoretically related to nurse performance, but theoretical plausibility should not be interpreted as empirical evidence of an appraisal effect. Similarly, studies examining education, leadership, work environment, professional development, or other determinants of nurse performance should not be interpreted as appraisal interventions unless they explicitly evaluated a formal performance‐appraisal process or system.

These findings identify a substantial evidence gap. Future research should use prospective and appropriately controlled designs to evaluate clearly defined appraisal interventions and should distinguish immediate appraisal‐related outcomes, such as perceived fairness, feedback usefulness, and measured performance, from more distal organizational or patient‐level outcomes. Where downstream outcomes are examined, studies should explicitly test the proposed pathways linking appraisal processes to those outcomes rather than assuming such relationships.

### 4.6. Research Gaps and Future Directions

Several important gaps emerged from the reviewed evidence. First, prospective and intervention‐based evidence evaluating formal nurse performance‐appraisal processes remains limited. Much of the literature is observational or methodological, which restricts conclusions regarding temporal relationships and causal effects. Future studies should therefore evaluate clearly defined appraisal interventions using prospective, quasiexperimental, or randomized designs where feasible, with appropriate comparison groups and sufficiently detailed reporting of implementation and context.

Second, greater conceptual clarity is needed regarding the distinction between nurse performance, professional competence, performance measurement, and formal performance appraisal. These constructs were operationalized differently across the included studies, contributing to substantial heterogeneity. Future research should define the construct being evaluated explicitly and ensure that selected measures correspond to the intended purpose of assessment.

Third, further development and validation of performance and competence measures should emphasize robust measurement properties and contextual applicability. Future instrument studies should provide adequate evidence concerning validity, reliability, measurement error, responsiveness where relevant, and external validation. Cross‐cultural or cross‐setting application of existing instruments should also be accompanied by appropriate evaluation rather than assuming that measurement properties are transferable across healthcare contexts.

Fourth, comparative evidence concerning different appraisal approaches is insufficient to establish the superiority of any single method or combination of methods. Direct comparisons of self‐assessment, supervisor assessment, multisource feedback, structured appraisal interviews, competence frameworks, and quantitative evaluation approaches could help determine their relative validity, feasibility, acceptability, and usefulness for specific appraisal purposes. Such studies should avoid assuming that more complex or multimethod approaches are inherently superior.

Fifth, evidence concerning outcomes directly attributable to formal performance appraisal requires substantial strengthening. Future studies should distinguish proximal appraisal‐related outcomes, such as perceived fairness, acceptability, feedback usefulness, and measured nurse performance, from more distal organizational and patient‐level outcomes. Claims regarding quality of care, patient safety, or patient outcomes should be based on studies that directly evaluate these outcomes in relation to clearly specified appraisal processes and adequately address alternative explanations.

Finally, the evidence‐informed conceptual framework proposed in this review requires empirical examination. Future longitudinal and intervention‐based studies could test specific relationships among measurement approaches, multilevel contextual determinants, formal appraisal processes, and appraisal‐related outcomes. Such research would help determine which relationships are reproducible across settings and whether particular pathways are causal, moderated, or mediated. Until such evidence is available, the framework should be regarded as an organizing conceptual synthesis rather than a validated predictive model.

### 4.7. Strengths

This review has several strengths. First, it provides an integrative synthesis of a heterogeneous evidence base by distinguishing four overlapping but conceptually distinct domains: tools and methods for measuring nurse performance or clinically relevant professional competence; determinants associated with measured performance or competence; formal performance‐appraisal processes and systems; and outcomes examined directly in relation to appraisal processes. This distinction reduces the risk of treating performance measurement, competence assessment, and formal organizational appraisal as interchangeable concepts.

Second, the review applied explicit eligibility criteria and a final study‐level eligibility audit to ensure that the primary synthesis was restricted to relevant primary empirical studies. Secondary literature was distinguished from the primary evidence base and retained only for contextual use where appropriate. Complete characteristics and domain classifications of the 40 included primary studies are provided in Supporting Table S1, thereby increasing the transparency of the evidence base underlying the synthesis.

Third, methodological quality was evaluated using design‐appropriate appraisal approaches rather than applying a single tool indiscriminately across heterogeneous study designs. MMAT 2018 [21] was used for applicable study categories, while measurement‐property, Delphi, decision‐analysis, model‐development, and framework‐development studies were considered using criteria appropriate to their methodological purposes. Study‐level appraisal judgments and methodological justifications are reported in Supporting File 3.

Fourth, the review maintained a conservative distinction between associations, study‐specific intervention findings, and causal conclusions. In particular, outcomes were classified as appraisal‐related only when they were examined directly in relation to a formal performance‐appraisal process or system. This approach reduced the risk of attributing broader professional, organizational, or patient‐level outcomes to performance appraisal without direct supporting evidence.

Finally, the evidence‐informed conceptual framework provides a structured representation of the relationships among measurement approaches, multilevel contextual determinants, formal appraisal processes, and appraisal‐related outcomes while explicitly avoiding assumptions of causality or mediation that were not established by the included evidence. The framework therefore provides an organizing basis for future empirical testing rather than presenting an unvalidated predictive model.

### 4.8. Study Limitations

This review has several limitations. First, the review protocol was not prospectively registered. Although the review was conducted and reported in accordance with PRISMA 2020 [20], the absence of prospective registration limits independent verification of whether methodological decisions changed during the review process.

Second, although the database‐specific search strategies and retrieval counts were retained and verified, the exact historical number of duplicate records removed and the exact number of records entering title‐and‐abstract screening could not be reliably reconstructed from the available documentation. These values were therefore not retrospectively estimated. This limitation affects the completeness of the numerical audit trail for the early stages of study selection, although the documented database searches retrieved 4527 records and the subsequent full‐text‐stage counts were reconciled across the manuscript, PRISMA flow diagram, and supporting information.

Third, the documented systematic search was limited to five bibliographic databases—PubMed, Scopus, Web of Science, CINAHL, and Embase—and eligible publications were restricted to English, Chinese, Turkish, Persian, and Korean. Relevant studies indexed exclusively in other databases or published in other languages may therefore have been missed. The language restriction may also have introduced language bias and should be considered when interpreting the geographical representation of the evidence.

Fourth, substantial clinical, conceptual, and methodological heterogeneity was present across the included studies. Definitions of nurse performance, professional competence, performance measurement, and formal performance appraisal varied, as did study designs, instruments, clinical settings, and outcomes. This heterogeneity precluded meaningful quantitative pooling and limits direct comparison of findings across studies.

Fifth, much of the evidence concerning determinants of measured nurse performance or competence was observational. Consequently, the reported relationships generally cannot establish temporal direction or causality. Similarly, only four included studies directly examined outcomes in relation to a formal performance‐appraisal process or system, limiting conclusions regarding the effectiveness of appraisal and preventing broad inference about downstream organizational or patient‐level outcomes.

Sixth, methodological quality varied across the evidence base, and incomplete reporting in some studies limited definitive appraisal of particular methodological domains. Although design‐appropriate appraisal approaches were used to contextualize interpretation, no formal assessment of reporting bias or certainty of the overall body of evidence was undertaken. No aggregate methodological‐quality score or post hoc quality‐based sensitivity analysis was performed.

Finally, the conceptual framework developed in this review was derived from the qualitative integration of heterogeneous evidence and has not been prospectively validated. The relationships depicted in the framework should therefore be interpreted as evidence‐informed conceptual connections rather than established causal, predictive, or mediational pathways. External validation and prospective testing are required before the framework can be used as an explanatory or predictive model.

### 4.9. Practical and Policy Implications

The findings have several implications for nursing management and healthcare organizations. First, organizations should distinguish between performance measurement and formal performance appraisal when designing or reviewing evaluation systems. Measurement instruments can provide structured information about selected dimensions of nurse performance or clinically relevant professional competence, whereas formal appraisal involves a broader organizational process that may include evaluation, feedback, managerial judgment, professional‐development planning, and other context‐specific components. Clarifying the intended purpose of evaluation is therefore important before selecting an appraisal approach or measurement instrument.

Second, the selection of performance measures should consider the construct being assessed, the measurement properties of the instrument, its feasibility, and its relevance to the clinical and organizational context. Instruments or frameworks developed for a particular nursing role, clinical unit, or healthcare system should not be assumed to perform equivalently in other contexts without appropriate evaluation or adaptation.

Third, interpretation of measured nurse performance should take account of relevant contextual factors. The reviewed evidence indicates that measured performance and competence are associated with individual and professional characteristics as well as organizational, managerial, work‐environment, and relational conditions. Performance information should therefore be interpreted within the context in which nursing practice occurs rather than being viewed exclusively as an indicator of individual capability or responsibility.

Fourth, formal appraisal processes should emphasize transparent criteria, clarity of purpose, appropriate preparation of evaluators, and meaningful feedback. These features are relevant to the acceptability and interpretability of appraisal processes; however, the present evidence does not establish that any single appraisal format or combination of methods is universally superior. Organizations should therefore adapt appraisal processes to their specific workforce, clinical context, available resources, and intended use while evaluating their implementation and outcomes.

Finally, policy and managerial decisions regarding performance appraisal should reflect the limited direct evidence concerning downstream effects. Although appraisal processes may support evaluation, feedback, and professional‐development activities, the current evidence base is insufficient to conclude that formal performance appraisal itself consistently improves patient safety, quality of care, or other patient‐level outcomes. Organizations implementing or redesigning appraisal systems should therefore incorporate systematic evaluation of both intended and unintended effects and avoid assuming benefits that have not been directly demonstrated.

## 5. Conclusion

This systematic review synthesized evidence from 40 primary empirical studies examining nurse performance evaluation across diverse healthcare settings. The evidence was heterogeneous and encompassed four overlapping domains: tools and methods for measuring nurse performance or clinically relevant professional competence; individual, professional, organizational, and work‐environment determinants associated with measured performance or competence; formal performance‐appraisal processes and systems; and outcomes examined directly in relation to appraisal processes.

A central finding of the review is the need to distinguish performance and competence measurement from formal performance appraisal. Measurement instruments and evaluation models provide information about specific dimensions of nurse performance or competence, whereas formal appraisal represents a broader organizational process that may incorporate measurement, managerial evaluation, feedback, and professional‐development activities. This distinction is important for both the interpretation of the existing literature and the design of future appraisal research.

The evidence further indicates that measured nurse performance is associated with factors operating across multiple individual, professional, organizational, managerial, and work‐environment levels. However, much of this evidence is observational, and these relationships should therefore be interpreted primarily as associations rather than established causal effects.

Evidence directly concerning the outcomes of formal performance‐appraisal processes was limited, with only four included studies explicitly examining outcomes in relation to an appraisal process or system. Consequently, the available evidence does not support broad conclusions that formal performance appraisal consistently improves nurse performance, organizational outcomes, quality of care, patient safety, or other patient‐level outcomes.

The conceptual framework developed in this review integrates measurement approaches, multilevel contextual determinants, formal appraisal processes, and appraisal‐related outcomes. It should be regarded as an evidence‐informed conceptual synthesis rather than a validated causal or predictive model. Future prospective and intervention‐based studies using clearly defined appraisal processes and well‐validated measures are needed to test the relationships proposed in the framework and to determine the effects of specific appraisal approaches across different healthcare contexts.

## Funding

This research received no specific grant from any funding agency in the public, commercial, or not‐for‐profit sectors.

## Conflicts of Interest

The authors declare no conflicts of interest.

## Supporting Information

Additional supporting information can be found online in the Supporting Information section.

## Supporting information


**Supporting Information 1** Supporting File 1. Complete database‐specific search strategies and retrieval counts for PubMed, Scopus, Web of Science, CINAHL, and Embase.

**Supporting Information 2** Supporting File 2. Full‐text retrieval information and reasons for exclusion following eligibility assessment.

**Supporting Information 3** Supporting File 3. Design‐appropriate methodological quality appraisal of the 40 included primary empirical studies.

**Supporting Information 4** Supporting Table S1. Characteristics, review‐domain classifications, key findings, and evidence/relationship types of the 40 included primary empirical studies.

**Supporting Information 5** PRISMA_2020_Checklist_Final.

## Data Availability

The data supporting the findings of this systematic review are available within the article and its supporting information. The supporting information includes the complete database‐specific search strategies, study‐selection information, study‐level methodological appraisal, and characteristics of the included primary studies.
