# Supplementary material for: Nurse Performance Evaluation in Healthcare: A Systematic Review of Measurement Approaches, Determinants, Appraisal Processes, and Outcomes
Source: J Nurs Manag. 2026 Oct 12;2026:9209587. doi: 10.1155/jonm/9209587 (PMC13659057; doi:10.1155/jonm/9209587)
Supplement: Supplementary file 1 [file JONM-2026-9209587-s001.docx]

**PRISMA 2020 Checklist**

**Completed for: Nurse Performance Evaluation in Healthcare: A Systematic Review of Measurement Approaches, Determinants, Appraisal Processes, and Outcomes**

Locations are based on the 46-page final manuscript supplied by the authors.

| **Section** | **Topic** | **Item #** | **Checklist item** | **Location where item is reported** |
| --- | --- | --- | --- | --- |
| TITLE | Title | 1 | Identify the report as a systematic review. | p. 1 (title) |
| ABSTRACT | Abstract | 2 | See the PRISMA 2020 for Abstracts checklist. | pp. 1–3 (Abstract) |
| INTRODUCTION | Rationale | 3 | Describe the rationale for the review in the context of existing knowledge. | pp. 3–5 (Introduction) |
| INTRODUCTION | Objectives | 4 | Provide an explicit statement of the objective(s) or question(s) the review addresses. | p. 5 (review aim and five review questions) |
| METHODS | Eligibility criteria | 5 | Specify the inclusion and exclusion criteria for the review and how studies were grouped for the syntheses. | pp. 7–10 (Eligibility Criteria); pp. 10, 12 (four synthesis domains) |
| METHODS | Information sources | 6 | Specify all databases, registers, websites, organisations, reference lists and other sources searched or consulted to identify studies. Specify the date when each source was last searched or consulted. | pp. 6–7 (PubMed, Scopus, Web of Science, CINAHL, Embase; final searches May 9, 2026) |
| METHODS | Search strategy | 7 | Present the full search strategies for all databases, registers and websites, including any filters and limits used. | pp. 6–7; Supplementary File 1 (complete database-specific strategies and retrieval counts) |
| METHODS | Selection process | 8 | Specify the methods used to decide whether a study met the inclusion criteria, including how many reviewers screened each record/report and whether they worked independently, and if applicable details of automation tools used. | p. 9 (independent assessment by two reviewers; consensus/third reviewer). No automation tool reported. |
| METHODS | Data collection process | 9 | Specify the methods used to collect data from reports, including how many reviewers collected data from each report, whether they worked independently, processes for obtaining or confirming data, and if applicable details of automation tools used. | p. 10 (standardized form; two independent reviewers; pilot testing, consensus/third reviewer, cross-checking). No automation tool reported. |
| METHODS | Data items | 10a | List and define all outcomes for which data were sought. Specify whether all results compatible with each outcome domain were sought, and if not, the methods used to decide which results to collect. | pp. 10, 12 (extracted appraisal/measurement approaches, determinants, appraisal-related outcomes and four evidence domains) |
| METHODS | Data items | 10b | List and define all other variables for which data were sought and describe any assumptions made about missing or unclear information. | p. 10 lists other extracted variables; assumptions about missing or unclear information were not separately reported. |
| METHODS | Study risk of bias assessment | 11 | Specify the methods used to assess risk of bias in the included studies, including details of the tool(s) used, how many reviewers assessed each study and whether they worked independently, and if applicable details of automation tools used. | p. 11 (MMAT 2018 for applicable designs; design-appropriate criteria for other designs; two independent reviewers); Supplementary File 3 |
| METHODS | Effect measures | 12 | Specify for each outcome the effect measure(s) used in the synthesis or presentation of results. | Not applicable: no meta-analysis or pooled effect-estimate synthesis was conducted; findings were synthesized qualitatively (pp. 11–12). |
| METHODS | Synthesis methods | 13a | Describe the processes used to decide which studies were eligible for each synthesis. | pp. 10, 12 (classification into four overlapping evidence domains; studies could contribute to more than one domain) |
| METHODS | Synthesis methods | 13b | Describe any methods required to prepare the data for presentation or synthesis, such as handling of missing summary statistics or data conversions. | No statistical data preparation or conversion was required/reported for the qualitative thematic synthesis. |
| METHODS | Synthesis methods | 13c | Describe any methods used to tabulate or visually display results of individual studies and syntheses. | pp. 14–23; Tables 1–5; Supplementary Table S1; Figure 2 |
| METHODS | Synthesis methods | 13d | Describe any methods used to synthesize results and provide a rationale for the choice(s). If meta-analysis was performed, describe the model(s), methods to identify heterogeneity, and software package(s) used. | pp. 11–12 (meta-analysis considered inappropriate because of heterogeneity; thematic synthesis informed by Thomas and Harden; NVivo version 12) |
| METHODS | Synthesis methods | 13e | Describe any methods used to explore possible causes of heterogeneity among study results. | No formal subgroup/meta-regression investigation was conducted; conceptual and methodological heterogeneity was addressed through qualitative domain-based synthesis (pp. 11–12). |
| METHODS | Synthesis methods | 13f | Describe any sensitivity analyses conducted to assess robustness of the synthesized results. | Not conducted. Explicitly reported on p. 11; see also pp. 21 and 35. |
| METHODS | Reporting bias assessment | 14 | Describe any methods used to assess risk of bias due to missing results in a synthesis (arising from reporting biases). | Not assessed. Explicitly stated on p. 11. |
| METHODS | Certainty assessment | 15 | Describe any methods used to assess certainty (or confidence) in the body of evidence for an outcome. | Not assessed. Explicitly stated on p. 11. |
| RESULTS | Study selection | 16a | Describe the results of the search and selection process, from the number of records identified in the search to the number of studies included in the review, ideally using a flow diagram. | pp. 13–14; Figure 1; Supplementary File 2. 4,527 records identified and 40 primary studies included; unreconstructable early-stage counts are transparently stated. |
| RESULTS | Study selection | 16b | Cite studies that might appear to meet the inclusion criteria, but which were excluded, and explain why they were excluded. | pp. 13–14 and Supplementary File 2 report full-text exclusion categories and counts. Individual near-eligible excluded studies are not separately cited. |
| RESULTS | Study characteristics | 17 | Cite each included study and present its characteristics. | pp. 15–23 and Tables 1–5; complete characteristics of all 40 studies in Supplementary Table S1 |
| RESULTS | Risk of bias in studies | 18 | Present assessments of risk of bias for each included study. | pp. 20–21 (summary); Supplementary File 3 (study-level judgments and methodological justifications) |
| RESULTS | Results of individual studies | 19 | For all outcomes, present for each study: (a) summary statistics for each group (where appropriate) and (b) an effect estimate and its precision, ideally using structured tables or plots. | Not applicable in the conventional effect-estimate sense because no quantitative meta-analysis was undertaken. Study-level key findings are summarized in Supplementary Table S1. |
| RESULTS | Results of syntheses | 20a | For each synthesis, briefly summarise the characteristics and risk of bias among contributing studies. | pp. 14–23; methodological-quality summary pp. 20–21; Supplementary Table S1 and Supplementary File 3 |
| RESULTS | Results of syntheses | 20b | Present results of all statistical syntheses conducted. If meta-analysis was done, present the summary estimate and its precision and measures of statistical heterogeneity. | Not applicable: no statistical synthesis or meta-analysis was conducted. |
| RESULTS | Results of syntheses | 20c | Present results of all investigations of possible causes of heterogeneity among study results. | No formal statistical investigation of heterogeneity was conducted. Conceptual/methodological heterogeneity is described throughout Results and Discussion (pp. 14–35). |
| RESULTS | Results of syntheses | 20d | Present results of all sensitivity analyses conducted to assess the robustness of the synthesized results. | Not applicable: no post hoc sensitivity analysis was conducted (pp. 21, 35). |
| RESULTS | Reporting biases | 21 | Present assessments of risk of bias due to missing results (arising from reporting biases) for each synthesis assessed. | Not assessed. Explicitly reported on pp. 21 and 35. |
| RESULTS | Certainty of evidence | 22 | Present assessments of certainty (or confidence) in the body of evidence for each outcome assessed. | Not assessed. Explicitly reported on pp. 21 and 35. |
| DISCUSSION | Discussion | 23a | Provide a general interpretation of the results in the context of other evidence. | pp. 23–33 (Discussion: Principal Findings and interpretation sections) |
| DISCUSSION | Discussion | 23b | Discuss any limitations of the evidence included in the review. | pp. 25–26 and 34–35 (Methodological Considerations; Study Limitations) |
| DISCUSSION | Discussion | 23c | Discuss any limitations of the review processes used. | pp. 34–35 (Study Limitations: no prospective registration; incomplete early screening audit trail; database/language restrictions; no reporting-bias/certainty assessment) |
| DISCUSSION | Discussion | 23d | Discuss implications of the results for practice, policy, and future research. | pp. 31–33 (Research Gaps and Future Directions); pp. 35–37 (Practical and Policy Implications) |
| OTHER INFORMATION | Registration and protocol | 24a | Provide registration information for the review, including register name and registration number, or state that the review was not registered. | p. 6: review was not prospectively registered in PROSPERO. |
| OTHER INFORMATION | Registration and protocol | 24b | Indicate where the review protocol can be accessed, or state that a protocol was not prepared. | p. 6: no prospectively registered review protocol was available. |
| OTHER INFORMATION | Registration and protocol | 24c | Describe and explain any amendments to information provided at registration or in the protocol. | Not applicable: the review was not prospectively registered and no prospective protocol was available. |
| OTHER INFORMATION | Support | 25 | Describe sources of financial or non-financial support for the review, and the role of the funders or sponsors in the review. | p. 38. Funding statement reports that the review received no specific grant from public, commercial, or not-for-profit funding agencies. |
| OTHER INFORMATION | Competing interests | 26 | Declare any competing interests of review authors. | p. 38: “The authors declare no conflict of interest.” |
| OTHER INFORMATION | Availability of data, code and other materials | 27 | Report which materials are publicly available and where they can be found (e.g., data collection forms, extracted data, analytic code, other review materials). | p. 38. Data Availability Statement reports that supporting data are available within the article and supplementary materials; Supplementary Files 1–3 and Supplementary Table S1 contain the search strategies, study-selection information, study-level methodological appraisal, and included-study characteristics. |

**Source:** Page MJ, McKenzie JE, Bossuyt PM, Boutron I, Hoffmann TC, Mulrow CD, et al. The PRISMA 2020 statement: an updated guideline for reporting systematic reviews. BMJ. 2021;372: n71. doi:10.1136/bmj. n71.

**Checklist completion note:** “Not assessed,” “Not applicable,” and “Not reported” are used deliberately where the supplied manuscript does not support a positive reporting claim. This avoids conflating methodological quality appraisal with PRISMA reporting-bias or certainty-of-evidence items.
