# Supplementary material for: Nurse Performance Evaluation in Healthcare: A Systematic Review of Measurement Approaches, Determinants, Appraisal Processes, and Outcomes
Source: J Nurs Manag. 2026 Oct 12;2026:9209587. doi: 10.1155/jonm/9209587 (PMC13659057; doi:10.1155/jonm/9209587)
Supplement: Supplementary file 2 [file JONM-2026-9209587-s002.docx]

**Final search date: May 9, 2026. The final searches were conducted on the same date across PubMed, Scopus, Web of Science, CINAHL, and Embase. The database-specific search strategies presented below correspond to the actual final searches conducted on May 9, 2026, and generated the retrieval counts reported in the review. They were not reconstructed from later exploratory reruns.**

**PubMed**

("Nurses"[Mesh] OR nurse*[tiab] OR "nursing staff"[tiab] OR "registered nurse*"[tiab]) AND ("Employee Performance Appraisal"[Mesh] OR "performance appraisal"[tiab] OR "performance apprais*"[tiab] OR "staff appraisal"[tiab] OR "employee performance appraisal"[tiab] OR "performance review"[tiab] OR "annual appraisal"[tiab] OR "annual review"[tiab] OR "performance feedback"[tiab] OR nurse performance[tiab] OR nursing performance[tiab] OR nursing performance assessment[tiab] OR nurse performance assessment[tiab] OR nursing performance measurement[tiab] OR nursing performance indicator*[tiab])

**816**

**Web of Science**

TS=(nurse* OR "nursing staff" OR "registered nurse*") AND TS=("performance appraisal" OR "performance apprais*" OR "staff appraisal" OR "employee performance appraisal" OR "performance review" OR "annual appraisal" OR "annual review" OR "performance feedback" OR "nurse performance" OR "nursing performance" OR "nursing performance assessment" OR "nurse performance assessment" OR "nursing performance measurement" OR "nursing performance indicator*")

**603**

**CINAHL**

(MH "Nurses+" OR MH "Registered Nurses" OR TI (nurse* OR "nursing staff" OR "registered nurse*") OR AB (nurse* OR "nursing staff" OR "registered nurse*")) AND (MH "Employee Performance Appraisal" OR TI ("performance appraisal" OR "performance apprais*" OR "staff appraisal" OR "employee performance appraisal" OR "performance review" OR "annual appraisal" OR "annual review" OR "performance feedback") OR AB ("performance appraisal" OR "performance apprais*" OR "staff appraisal" OR "employee performance appraisal" OR "performance review" OR "annual appraisal" OR "annual review" OR "performance feedback"))

**832**

**Embase**

('nurse'/exp OR 'nursing staff'/exp OR nurse*:ti,ab OR 'nursing staff':ti,ab OR 'registered nurse*':ti,ab) AND ('employee performance appraisal'/exp OR 'performance appraisal':ti,ab OR 'performance apprais*':ti,ab OR 'staff appraisal':ti,ab OR 'employee performance appraisal':ti,ab OR 'performance review':ti,ab OR 'annual appraisal':ti,ab OR 'annual review':ti,ab OR 'performance feedback':ti,ab)

**825**

**Scopus**

TITLE-ABS-KEY (nurse* OR "nursing staff" OR "registered nurse*") AND TITLE-ABS-KEY ("performance appraisal" OR "performance apprais*" OR "staff appraisal" OR "employee performance appraisal" OR "performance review" OR "annual appraisal" OR "annual review" OR "performance feedback" OR "nurse performance" OR "nursing performance" OR "nursing performance assessment" OR "nurse performance assessment" OR "nursing performance measurement" OR "nursing performance indicator*")

**1451**
