# Supplementary material for: Nurse Performance Evaluation in Healthcare: A Systematic Review of Measurement Approaches, Determinants, Appraisal Processes, and Outcomes
Source: J Nurs Manag. 2026 Oct 12;2026:9209587. doi: 10.1155/jonm/9209587 (PMC13659057; doi:10.1155/jonm/9209587)
Supplement: Supplementary file 3 [file JONM-2026-9209587-s003.docx]

Following title and abstract screening, 328 reports were sought for full-text retrieval. Twelve reports could not be retrieved, leaving 316 reports that were assessed against the predefined eligibility criteria. Following the final eligibility audit, 276 reports were excluded after full-text assessment. The reasons for exclusion are summarized in Table S2.

Reports not retrieved (n = 12): Full-text reports that could not be obtained for eligibility assessment despite available retrieval attempts.

**Table S2. Reasons for Exclusion Following Full-Text Assessment**

| **Reason for exclusion** | **Number of reports (n)** | **Description** |
| --- | --- | --- |
| **Outside the predefined conceptual scope** | **122** | Reports that did not meet the predefined conceptual scope of nurse performance appraisal, performance measurement, determinants examined in relation to explicitly measured nurse performance or clinically relevant competence, or outcomes directly related to an appraisal process or system. |
| **No eligible empirical data** | **76** | Reports that did not provide original empirical data meeting the review eligibility criteria. Publications classified separately as reviews, editorials, or commentaries were not included in this category. |
| **Ineligible or non-nursing population** | **47** | Studies involving nursing students, physicians, allied health professionals, or other populations that did not meet the predefined population criterion, including mixed professional populations without separately extractable nurse-specific data. |
| **Reviews, editorials, or commentaries** | **30** | Secondary or non-primary publications excluded from the primary systematic synthesis. Relevant review articles could be used only as contextual sources in the Introduction or Discussion and were not counted among the included primary empirical studies or incorporated into the primary thematic synthesis. |
| **Duplicate publication identified during final eligibility assessment** | **1** | A duplicate publication representing an already included study was identified during the final eligibility audit and was not counted as a separate primary study. |
| **Total excluded after full-text assessment** | **276** | — |

Following full-text retrieval and eligibility assessment, 40 primary empirical studies met the eligibility criteria and were included in the qualitative synthesis. Two relevant review articles identified during the search were retained only as contextual sources for interpretation and comparison and were not counted among the 40 included primary studies, subjected to primary-study data extraction or methodological appraisal, or incorporated into the primary thematic synthesis. Complete characteristics of the 40 included primary studies are presented in Supplementary Table S1.
