# Supplementary material for: Nurse Performance Evaluation in Healthcare: A Systematic Review of Measurement Approaches, Determinants, Appraisal Processes, and Outcomes
Source: J Nurs Manag. 2026 Oct 12;2026:9209587. doi: 10.1155/jonm/9209587 (PMC13659057; doi:10.1155/jonm/9209587)
Supplement: Supplementary file 4 [file JONM-2026-9209587-s004.docx]

**Supplementary File 3. Revised Design-Appropriate Methodological Quality Appraisal of the 40 Primary Empirical Studies**

**Appraisal approach.** Studies were appraised using a design-appropriate approach. Empirical studies fitting the Mixed Methods Appraisal Tool (MMAT) 2018 categories were assessed with the relevant MMAT criteria. Instrument-development and psychometric studies were assessed using COSMIN-informed measurement-property domains. The Delphi study was assessed using CREDES-informed criteria. Decision-analysis, performance-model, and framework-development studies were assessed with a structured design-specific appraisal focused on methodological transparency, appropriateness of methods, validation, and limitations. No overall numerical quality score was calculated.

**Judgment codes.** Y = criterion adequately addressed; N = criterion not adequately addressed; CT = cannot tell because reporting available for appraisal was insufficient for a defensible judgment; NA = not applicable. CT was preferred to an unsupported positive judgment when reporting was incomplete.

# A. Studies appraised with MMAT 2018

**MMAT columns:** SQ1 = clear research questions; SQ2 = data collected allow the research questions to be addressed. C1-C5 are the five design-specific MMAT criteria for the assigned category.

| **Study** | **Design** | **MMAT category** | **SQ1** | **SQ2** | **C1** | **C2** | **C3** | **C4** | **C5** | **Brief justification** |
| --- | --- | --- | --- | --- | --- | --- | --- | --- | --- | --- |
| Chen et al. (2019) | Longitudinal observational | MMAT: Quantitative non-randomized | Y | Y | CT | Y | CT | CT | Y | Longitudinal design was appropriate for examining temporal associations, but available reporting was insufficient to confirm representativeness, completeness of follow-up, and control of confounding. |
| Sharma & Dhar (2016) | Cross-sectional | MMAT: Quantitative descriptive | Y | Y | Y | CT | Y | CT | Y | Clear association/mediation question and measured performance outcome; representativeness and non-response bias were not sufficiently documented for an unequivocal positive judgment. |
| Tran et al. (2018) | Cross-sectional/SEM | MMAT: Quantitative descriptive | Y | Y | Y | CT | Y | CT | Y | Questionnaire survey of hospital nurses with structural equation modeling; measurements and analyses were aligned with the research question, while representativeness and non-response bias remained incompletely documented. |
| Sarıköse & Göktepe (2022) | Cross-sectional | MMAT: Quantitative descriptive | Y | Y | Y | CT | Y | CT | Y | Use of a nursing job-performance measure supported the research question; sampling representativeness and potential non-response bias could not be fully established from the available report. |
| Awases et al. (2013) | Quantitative descriptive survey | MMAT: Quantitative descriptive | Y | Y | Y | Y | CT | CT | Y | A random sample of 180 professional nurses from six hospitals in three regions was reported, supporting the sampling strategy; response/non-response details were not sufficient for a definitive bias judgment. |
| Al-Makhaita et al. (2014) | Comparative cross-sectional | MMAT: Quantitative descriptive | Y | Y | Y | CT | Y | CT | Y | Comparative design and performance measurement were appropriate to the stated objective; representativeness and non-response bias were insufficiently reported. |
| Chairina et al. (2019) | Cross-sectional | MMAT: Quantitative descriptive | Y | Y | CT | CT | CT | CT | Y | The study addressed determinants of nurse performance, but available reporting did not permit confident judgments on sampling, measurement validity, or non-response bias. |
| Youssif et al. (2017) | Descriptive correlational/cross-sectional | MMAT: Quantitative descriptive | Y | Y | Y | CT | Y | CT | Y | The appraisal, empowerment, and job-satisfaction measures addressed the correlational objective; representativeness and non-response bias were not fully established. |
| Ali et al. (2024) | Quasi-experimental | MMAT: Quantitative non-randomized | Y | Y | CT | Y | CT | CT | Y | The intervention and outcomes were directly relevant to appraisal fairness; lack of randomization makes confounding important, and available reporting was insufficient to confirm full control of confounders and completeness of outcome data. |
| Nikpeyma et al. (2014) | Descriptive qualitative | MMAT: Qualitative | Y | Y | Y | Y | Y | Y | CT | Purposive sampling, focus groups to saturation, and framework analysis were explicitly described and coherent with the qualitative objective; researcher influence/reflexivity was not sufficiently reported. |
| Asiamah et al. (2019) | Cross-sectional | MMAT: Quantitative descriptive | Y | Y | CT | CT | CT | CT | Y | The study linked education/training with a measured nursing-performance outcome, but sampling, measurement quality, and non-response reporting were insufficient for uniformly positive judgments. |
| Cho & Han (2018) | Multilevel cross-sectional | MMAT: Quantitative descriptive | Y | Y | Y | CT | Y | CT | Y | The multilevel design and work-environment/performance measures were suited to the question; representativeness and non-response bias were not fully documented. |
| Widyana & Handiyani (2019) | Cross-sectional | MMAT: Quantitative descriptive | Y | Y | CT | CT | CT | CT | Y | The study examined self-efficacy/organizing competency in relation to performance; details needed to judge sampling, measurement properties, and non-response bias were incomplete. |
| Soares et al. (2019) | Descriptive evaluation study | MMAT: Quantitative descriptive | Y | Y | CT | CT | Y | CT | CT | Competence-based performance evaluation was directly relevant, but available reporting was insufficient to establish representativeness, non-response bias, and the full appropriateness of statistical analysis. |
| Jaber et al. (2024) | Cross-sectional descriptive comparative | MMAT: Quantitative descriptive | Y | Y | Y | CT | Y | CT | Y | A performance-appraisal efficacy questionnaire and comparative analysis addressed the research objective; representativeness and non-response bias could not be fully verified. |
| Top (2013) | Cross-sectional | MMAT: Quantitative descriptive | Y | Y | CT | CT | CT | CT | Y | Organizational variables were examined against nurse job performance, but available reporting did not support uniformly positive judgments for sampling, measurement, and non-response domains. |
| Chen et al. (2023) | Cross-sectional | MMAT: Quantitative descriptive | Y | Y | Y | CT | Y | CT | Y | Registered nurses' emergency-care competence was explicitly measured; the single regional-hospital context limits representativeness, and non-response bias was not sufficiently documented. |
| Al-Hawary & Banat (2017) | Cross-sectional | MMAT: Quantitative descriptive | Y | Y | CT | CT | CT | CT | Y | Motivation and job performance were directly examined, but methodological details available for appraisal were insufficient for confident judgments on representativeness, measurement quality, and non-response. |
| Fujino et al. (2015) | Cross-sectional | MMAT: Quantitative descriptive | Y | Y | Y | CT | Y | CT | Y | Performance and explanatory variables were explicitly measured and analyzed; representativeness and non-response bias were incompletely documented. |
| Kim et al. (2015) | Cross-sectional | MMAT: Quantitative descriptive | Y | Y | Y | CT | Y | CT | Y | Clinical competence and professional quality-of-life measures were appropriate to the association question; representativeness and non-response bias could not be fully established. |
| Kamati et al. (2014) | Cross-sectional | MMAT: Quantitative descriptive | Y | Y | CT | CT | CT | CT | CT | The study addressed factors influencing registered nurses' performance, but the available report did not provide enough detail for several MMAT domains. |
| Bhatti et al. (2018) | Cross-sectional/mediation analysis | MMAT: Quantitative descriptive | Y | Y | Y | CT | Y | CT | Y | Job resources, work engagement, and performance were measured in a mediation model; representativeness and non-response bias remained insufficiently documented. |
| Salanova et al. (2011) | Cross-sectional/mediation model | MMAT: Quantitative descriptive | Y | Y | Y | CT | Y | CT | Y | The measured leadership, self-efficacy, engagement, and performance constructs matched the analytical model; representativeness and non-response bias were not fully verifiable. |
| Geyer et al. (2018) | Cross-sectional | MMAT: Quantitative descriptive | Y | Y | CT | CT | Y | CT | Y | Work performance and caring-related constructs were measured, but sampling representativeness and non-response reporting were insufficiently clear. |
| Shen et al. (2022) | Cross-sectional | MMAT: Quantitative descriptive | Y | Y | Y | CT | Y | CT | Y | A nurse work-performance questionnaire was used with individual/workplace factors; representativeness and non-response bias were not fully established. |
| Bigdeli et al. (2019) | Randomized controlled trial | MMAT: Randomized controlled trial | Y | Y | CT | CT | CT | CT | CT | The study was reported as randomized and directly evaluated appraisal interviews, but the available report was insufficient to verify sequence generation, baseline comparability, completeness, blinding, and adherence in enough detail for positive judgments. |

# B. Studies requiring alternative design-appropriate appraisal

**Alternative-appraisal columns:** A1-A5 are design-specific domains. For COSMIN-informed studies: A1 instrument-development/content basis; A2 validity evidence; A3 internal consistency/measurement structure; A4 reliability/measurement error; A5 construct/criterion validity and interpretability. For Delphi/model/framework studies, A1-A5 correspond to rationale/objective, process/input transparency, methodological appropriateness, validation/robustness, and transferability/limitations, respectively.

| **Study** | **Design** | **Appraisal approach** | **A1** | **A2** | **A3** | **A4** | **A5** | **Brief justification** |
| --- | --- | --- | --- | --- | --- | --- | --- | --- |
| Lin et al. (2010) | Instrument development and psychometric evaluation | COSMIN-informed measurement-property appraisal | Y | Y | Y | CT | Y | Scale development and psychometric evaluation were explicit; evidence supported content/construct evaluation and internal consistency, while reliability/measurement-error reporting could not be fully verified from the available report. |
| Crossley (2015) | Instrument development and validation | COSMIN-informed measurement-property appraisal | Y | Y | Y | CT | Y | Development and validation of a multisource-feedback instrument were explicit; available reporting supported validity and consistency evidence, with incomplete information for some reliability/measurement-error domains. |
| Holanda et al. (2018) | Instrument development and validation | COSMIN-informed measurement-property appraisal | Y | Y | Y | CT | Y | The study explicitly created and validated an emergency-nurse competence instrument; some measurement-property domains were supported, while reliability/measurement error could not be fully verified. |
| Kahya & Oral (2018a) | Instrument development | COSMIN-informed measurement-property appraisal | Y | Y | CT | CT | Y | The study developed a clinical nurse performance tool with task/contextual items; full reporting was insufficient to verify all reliability and internal-consistency domains. |
| Lee (2016) | Instrument development | COSMIN-informed measurement-property appraisal | Y | Y | CT | CT | Y | A unit-specific appraisal tool was developed for PACU nurses; content/construct development was evident, but complete psychometric evidence could not be verified from the available report. |
| Nuryanti et al. (2017) | Instrument development | COSMIN-informed measurement-property appraisal | Y | CT | CT | CT | CT | A web-based appraisal instrument was developed, but the available report did not provide enough detail to establish the breadth of validity and reliability testing. |
| Nuritasari et al. (2020) | Instrument development | COSMIN-informed measurement-property appraisal | Y | CT | CT | CT | CT | The study developed a self-efficacy-based appraisal instrument; reporting available for appraisal was insufficient to confirm comprehensive psychometric testing. |
| Li et al. (2025) | Delphi study | CREDES-informed Delphi appraisal | Y | Y | Y | CT | CT | The Delphi approach was justified for competency-index development and expert consensus was reported; however, the available reporting was insufficient to establish a priori consensus thresholds and attrition/response characteristics with confidence. |
| Cheng et al. (2021) | Decision-analysis study | Structured decision/model appraisal | Y | Y | Y | CT | CT | The hybrid MADM model was clearly linked to competency evaluation; model specification and criteria were described, while external validation and sensitivity/robustness assessment were not clearly established. |
| Kahya & Oral (2018b) | Performance-criteria framework study | Structured methodological appraisal | Y | Y | Y | CT | CT | The study explicitly identified and weighted clinical nurse performance criteria; transparency of criterion generation was adequate at a broad level, while evidence regarding external validation and reproducibility was insufficiently reported. |
| Gedük et al. (2023) | Development and implementation study | Structured instrument/implementation appraisal | Y | Y | Y | CT | CT | Development and implementation of a structured appraisal tool were reported; broader validation, implementation fidelity, and transferability beyond the study setting were insufficiently established. |
| Sullivan et al. (2021) | Framework development and pilot implementation | Structured framework/pilot appraisal | Y | Y | Y | CT | CT | The Q-PACE framework had a clear rationale and was piloted in a major healthcare organization; the mixed nursing/midwifery context was explicit, but rigorous outcome validation and transferability require further evidence. |
| Osman et al. (2011) | Quantitative modeling (DEA) | Structured model appraisal | Y | Y | Y | CT | CT | DEA was explicitly used for relative nurse-performance evaluation; the modeling approach was aligned with the objective, but external validation and sensitivity to modeling assumptions were not fully established. |
| Yang (2022) | Performance-model development | Structured model appraisal | Y | Y | Y | CT | CT | A Balanced Scorecard-based multidimensional model was developed with a clear evaluative purpose; independent validation and robustness/sensitivity evidence were not sufficiently documented. |

# Interpretive summary

The reappraisal did not support uniformly positive methodological judgments. Across the observational evidence, the most recurrent uncertainties concerned representativeness, non-response bias, completeness of outcome reporting, and control of confounding. The qualitative appraisal was generally coherent with the study aim but had limited reporting on reflexivity. For the randomized and quasi-experimental studies, reporting available for appraisal did not permit all risk-of-bias domains to be judged positively. Instrument-development studies showed evidence of structured development and validity assessment, but the breadth of reliability, measurement-error, and external-validation evidence varied or was incompletely reported. Delphi, decision-analysis, and model/framework studies were therefore not forced into an inappropriate MMAT category.

# Appraisal references

Hong QN, Pluye P, Fàbregues S, et al. Mixed Methods Appraisal Tool (MMAT), version 2018: User guide. McGill University; 2018.

Mokkink LB, de Vet HCW, Prinsen CAC, et al. COSMIN Risk of Bias checklist for systematic reviews of Patient-Reported Outcome Measures. Qual Life Res. 2018;27:1171-1179.

Jünger S, Payne SA, Brine J, Radbruch L, Brearley SG. Guidance on Conducting and REporting DElphi Studies (CREDES) in palliative care. Palliat Med. 2017;31(8):684-706.

**Note on appraisal judgments:** CT indicates that the information reported in the published article was insufficient to support a definitive “Yes” or “No” judgment for the corresponding criterion or domain. In such cases, a “Cannot tell” judgment was assigned rather than assuming that the criterion had been adequately met. No overall numerical quality score was calculated.
