# Supplementary material for: Nurse Performance Evaluation in Healthcare: A Systematic Review of Measurement Approaches, Determinants, Appraisal Processes, and Outcomes
Source: J Nurs Manag. 2026 Oct 12;2026:9209587. doi: 10.1155/jonm/9209587 (PMC13659057; doi:10.1155/jonm/9209587)
Supplement: Supplementary file 5 [file JONM-2026-9209587-s005.docx]

**Supplementary Table S1. Characteristics of the 40 Primary Empirical Studies Included in the Systematic Review of Nurse Performance Appraisal**

Evidence domains: Tool/Measurement = development, validation, or application of tools, scales, criteria, or models used to assess nurse performance or clinically relevant professional competence; Determinant = individual, professional, organizational, or work-environment factors examined in relation to explicitly measured nurse performance or clinical competence; Appraisal Process/System = formal performance appraisal processes, systems, interviews, feedback, implementation, or appraisal frameworks; Appraisal-related Outcome = outcomes examined in direct relation to a performance appraisal process or system. Studies may contribute to more than one domain.

| **First author (Year)** | **Country** | **Study design** | **Setting** | **Appraisal / performance measure** | **Review domain** | **Main focus** | **Key findings** | **Evidence / relationship type** |
| --- | --- | --- | --- | --- | --- | --- | --- | --- |
| Chen et al. (2019) | Taiwan | Longitudinal study | Emergency department | Job performance assessment | Determinant | Work-related factors influencing nurse job performance | Work-related factors were significantly associated with nurses' job performance over time. | Longitudinal association |
| Sharma & Dhar (2016) | India | Cross-sectional | Hospital | Job performance questionnaire | Determinant | Organizational factors and affective commitment | Affective commitment mediated the relationship between workplace factors and nurse performance. | Mediation analysis (cross-sectional) |
| Tran et al. (2018) | Vietnam | Cross-sectional | Healthcare organizations | Self-report performance questionnaire | Determinant | Workplace relationships and nurse job performance | Higher-quality workplace relationships were associated with better nurse job performance. | Cross-sectional association |
| Sarıköse & Göktepe (2022) | Türkiye | Cross-sectional | Hospital | Nursing Job Performance Scale | Determinant | Individual, professional, and work-environment characteristics | Individual characteristics and the work environment were associated with nursing job performance. | Cross-sectional association |
| Awases et al. (2013) | Namibia | Cross-sectional | Healthcare facilities | Performance assessment | Determinant | Factors affecting professional nurse performance | Organizational support, motivation, and resources were identified as factors related to performance. | Cross-sectional descriptive/associational evidence |
| Al-Makhaita et al. (2014) | Saudi Arabia | Comparative cross-sectional | Primary and secondary healthcare | Job performance questionnaire | Determinant | Nurse performance across healthcare levels | Nurse performance varied according to healthcare level and work-related characteristics. | Comparative cross-sectional association |
| Chairina et al. (2019) | Indonesia | Cross-sectional | Accredited private hospitals | Performance evaluation questionnaire | Determinant | Determinants of nurse performance | Leadership, motivation, and organizational support were associated with nurse performance. | Cross-sectional association |
| Youssif et al. (2017) | Egypt | Cross-sectional | Hospital | Performance appraisal tool | Appraisal Process/System + Appraisal-related Outcome | Performance appraisal, job satisfaction, and empowerment | Perceptions of appraisal were associated with job satisfaction and empowerment. | Cross-sectional association with appraisal-related outcomes |
| Ali et al. (2024) | Egypt | Quasi-experimental | Hospital | Nurse-manager appraisal training program | Appraisal Process/System + Appraisal-related Outcome | Fairness of performance appraisal | Training was associated with improved perceptions of appraisal fairness and collaboration. | Quasi-experimental intervention effect |
| Nikpeyma et al. (2014) | Iran | Qualitative study | Clinical settings | Interviews | Appraisal Process/System | Problems in nurse performance appraisal | Participants described appraisal systems as subjective and insufficiently transparent. | Qualitative descriptive evidence |
| Asiamah et al. (2019) | Ghana | Cross-sectional | Healthcare organizations | Questionnaire with nursing performance outcome | Determinant | Education and in-service training | Education and in-service training were positively associated with nursing performance. | Cross-sectional association |
| Cho & Han (2018) | South Korea | Multilevel cross-sectional | Hospitals | Work-environment and nursing-performance measures | Determinant | Work environment, health-promoting behaviors, and nursing performance | More favorable work environments were associated with better nursing performance. | Multilevel cross-sectional association |
| Widyana & Handiyani (2019) | Indonesia | Cross-sectional | Hospitals | Questionnaire with nursing-performance measure | Determinant | Self-efficacy and organizing competency | Self-efficacy and managerial/organizing competency were associated with nursing performance. | Cross-sectional association |
| Lin et al. (2010) | Taiwan | Instrument development and psychometric evaluation | Public health services | Public Health Nurse Professional Competency Scale | Tool/Measurement | Professional competency measurement | The scale demonstrated acceptable psychometric properties for assessing professional competency in public health nurses. | Instrument development and psychometric validation |
| Crossley (2015) | United Kingdom | Instrument development and validation | Clinical practice | Multisource Feedback instrument | Tool/Measurement + Appraisal Process/System | Multisource appraisal for nurses | The instrument supported a structured multisource approach to nurse appraisal. | Instrument development/validation; structured multisource appraisal |
| Li et al. (2025) | China | Delphi study | Pain management services | Postcompetency Index System | Tool/Measurement | Competency index for pain resource nurses | Expert consensus produced a structured competency index intended for selection, training, and assessment. | Delphi consensus / competency-index development |
| Holanda et al. (2018) | Brazil | Instrument development and validation | Emergency departments | Professional competence assessment instrument | Tool/Measurement | Assessment of emergency nurses' professional competence | The instrument demonstrated evidence of validity for assessing emergency nursing competence. | Instrument development and validation |
| Cheng et al. (2021) | Taiwan | Decision-analysis study | Clinical education / hospital practice | Hybrid MADM competency-evaluation model | Tool/Measurement | Competency evaluation of newly graduated nurses | The model was used to identify competency gaps and support individualized improvement planning. | Decision-analysis / model-based evaluation |
| Kahya & Oral (2018a) | Türkiye | Instrument development | Hospital | Clinical Nurse Performance Measurement Tool | Tool/Measurement | Measurement of clinical nurse performance | The instrument integrated task and contextual dimensions of clinical nurse performance. | Instrument development |
| Kahya & Oral (2018b) | Türkiye | Descriptive study | Hospital | Performance criteria framework | Tool/Measurement | Identification and weighting of clinical nurse performance criteria | The study proposed a structured set of criteria for evaluating clinical nurse performance. | Descriptive criteria development/weighting |
| Lee (2016) | South Korea | Instrument development | Post-anesthesia care unit | Performance appraisal tool | Tool/Measurement + Appraisal Process/System | Development of a unit-specific appraisal tool | The tool was developed to support structured evaluation of PACU nurses. | Instrument development; structured appraisal tool |
| Soares et al. (2019) | Brazil | Descriptive study | Hospital | Competency-based performance evaluation | Tool/Measurement + Appraisal Process/System | Competence-based performance evaluation | Competency-based evaluation was used to identify strengths and development needs in nursing practice. | Descriptive application of competence-based appraisal |
| Gedük et al. (2023) | Türkiye | Development and implementation study | State hospital | Nurse Performance Appraisal Tool | Tool/Measurement + Appraisal Process/System | Development and implementation of appraisal tools | The study reported development and implementation of a structured nurse performance appraisal approach. | Instrument development and implementation |
| Sullivan et al. (2021) | Qatar | Framework development and pilot implementation | Major healthcare organization; nursing and midwifery workforce | Q-PACE (Qatar Practice, Appraisal, Competence and Education) competence framework | Tool/Measurement + Appraisal Process/System | Development and piloting of an integrated framework linking clinical practice, appraisal, competence, and education | The Q-PACE framework integrated previously fragmented competency-confirmation and appraisal activities into a unified process for new and existing staff; the mixed nursing/midwifery population is reported explicitly. | Framework development and pilot implementation |
| Jaber et al. (2024) | Saudi Arabia | Cross-sectional descriptive comparative study | Tertiary hospital | Performance appraisal efficacy questionnaire | Appraisal Process/System + Appraisal-related Outcome | Nurses' views of appraisal-system efficacy and work outcomes | Nurses' perceptions of appraisal effectiveness and efficiency varied across nursing roles and were related to work outcomes. | Cross-sectional descriptive-comparative association with appraisal-related outcomes |
| Top (2013) | Türkiye | Cross-sectional | Public and private hospitals | Organizational variables and job-performance questionnaire | Determinant | Organizational factors influencing nurse job performance | Workload, technological support, managerial practices, and work environment were related to nurse job performance. | Cross-sectional association |
| Nuryanti et al. (2017) | Indonesia | Instrument development study | Inpatient hospital | Web-based nurse performance appraisal instrument | Tool/Measurement + Appraisal Process/System | Development of a web-based appraisal instrument | A web-based instrument was developed to support more standardized and efficient nurse performance appraisal. | Instrument development / web-based appraisal system |
| Bigdeli et al. (2019) | Iran | Randomized controlled trial | Neonatal intensive care units | Performance appraisal interview | Appraisal Process/System + Appraisal-related Outcome | Effect of appraisal interviews on nurses' performance | Structured appraisal interviews were associated with improved nurse performance compared with routine evaluation. | Randomized controlled intervention effect |
| Nuritasari et al. (2020) | Indonesia | Instrument development | Hospital | Self-efficacy-based performance appraisal instrument | Tool/Measurement + Appraisal Process/System | Development of a nurse appraisal instrument | Self-efficacy was incorporated into a structured nurse performance appraisal instrument. | Instrument development |
| Chen et al. (2023) | Taiwan | Cross-sectional study | Regional hospital | Self-assessed interprofessional emergency care competence scale following gamified CPR training | Tool/Measurement | Assessment of registered nurses’ emergency-care competence and factors associated with measured competence | Among 194 registered nurses, perceived usefulness and other technology-acceptance factors were positively associated with self-assessed emergency-care competence; the study is included as competence measurement rather than as evidence of a formal appraisal intervention. | Cross-sectional association (competence outcome) |
| Osman et al. (2011) | Lebanon | Quantitative modeling study | Intensive care unit | Data Envelopment Analysis (DEA) | Tool/Measurement | Relative nurse performance evaluation | DEA was used to differentiate relative nurse performance and identify efficiency variation. | Quantitative modeling / relative performance evaluation |
| Yang (2022) | China | Model development study | Community health service centers | Balanced Scorecard-based model | Tool/Measurement + Appraisal Process/System | Nursing performance evaluation model | A Balanced Scorecard-based model was developed for multidimensional nursing performance evaluation. | Performance-model development |
| Al-Hawary & Banat (2017) | Jordan | Cross-sectional | Private hospitals | Motivation and job-performance questionnaire | Determinant | Motivation and nurse job performance | Higher motivation was associated with better nurse job performance. | Cross-sectional association |
| Fujino et al. (2015) | Japan | Cross-sectional | Hospital | Nursing performance assessment | Determinant | Emotional intelligence, work experience, and nursing performance | Emotional intelligence and professional experience were positively associated with nursing performance. | Cross-sectional association |
| Kim et al. (2015) | South Korea | Cross-sectional | Hospital | Professional Quality of Life Scale and Clinical Competency Assessment | Determinant | Professional quality of life and clinical competence | Professional quality-of-life dimensions were associated with measured clinical competence among nurses. | Cross-sectional association |
| Kamati et al. (2014) | Namibia | Cross-sectional | National referral hospital | Structured questionnaire | Determinant | Factors influencing registered nurses' performance | Training, staffing, managerial factors, and availability of resources were identified as factors influencing registered nurses' performance. | Cross-sectional descriptive/associational evidence |
| Bhatti et al. (2018) | Malaysia | Cross-sectional | Hospital | Job resources, work engagement, and performance measures | Determinant | Job resources, work engagement, and performance | Work engagement mediated the association between job resources and nurses' job performance. | Mediation analysis (cross-sectional) |
| Salanova et al. (2011) | Portugal | Cross-sectional | Hospital | Self-report leadership, self-efficacy, engagement, and performance measures | Determinant | Transformational leadership and extra-role performance | Transformational leadership was associated with extra-role performance through self-efficacy and work engagement. | Mediation analysis (cross-sectional) |
| Geyer et al. (2018) | South Africa | Cross-sectional | Hospital | Intrapersonal-characteristics and work-performance measures | Determinant | Intrapersonal characteristics, work performance, and caring behaviors | Positive intrapersonal characteristics were associated with better work performance and caring behaviors. | Cross-sectional association |
| Shen et al. (2022) | China | Cross-sectional | Hospital | Schwirian’s Six-Dimension Scale (Six-D Scale) | Determinant | Work performance among male nurses | Individual and workplace factors were significantly associated with work performance among male nurses. | Cross-sectional association |

Note. This revised table reflects the eligibility re-audit undertaken in response to reviewer concerns regarding conceptual scope and consistency of inclusion criteria. The review scope follows the article title and research questions by distinguishing tools/measurement, determinants of measured nurse performance, appraisal processes/systems, and appraisal-related outcomes. Secondary reviews used only for contextual comparison, non-eligible populations or concepts, and duplicate records are not counted among the 40 primary studies. Clinical competence studies were retained only when competence was explicitly assessed as an evaluative domain or proxy of professional nursing performance in practicing nurses. Mixed nursing/midwifery evidence was retained only when it directly informed an appraisal/competence framework relevant to the review scope and is identified transparently in the table. The “Evidence / relationship type” column provides an explicit audit trail of the type of evidence contributed by each study (e.g., descriptive, cross-sectional association, longitudinal association, mediation analysis, intervention effect, instrument development/validation, consensus, or model/framework development); these labels describe the analytical relationship or methodological contribution and do not imply causality unless supported by the original study design.
